# Soil-Gradient-Derived Bacterial Synthetic Communities Enhance Drought Tolerance in *Quercus pubescens* and *Sorbus domestica* Seedlings

**DOI:** 10.3390/plants14111659

**Published:** 2025-05-29

**Authors:** Ivan Aleksieienko, Mariana Fernandes Hertel, Jérôme Reilhan, Marie de Castro, Bertrand Légeret, Halley Caixeta Oliveira, Ilja M. Reiter, Catherine Santaella

**Affiliations:** 1 Aix Marseille Univ, CEA, CNRS, BIAM, LEMiRE, ECCOREV FR 3098, F-13108 Saint Paul Lez Durance, France; mariana.fernandes-hertel@cea.fr; 2 State University of Londrina, Department of Animal and Plant Biology, Londrina 86057-970, Paraná, Brazil; halley@uel.br; 3 ONF, PNRGF Cadarache, F-13108 Saint Paul Lez Durance, France; jerome.reilhan@onf.fr (J.R.); marie.de-castro@onf.fr (M.d.C.); 4 Aix Marseille Univ, CEA, CNRS, BIAM, EBMP, F-13108 Saint Paul Lez Durance, France; bertrand.legeret@cea.fr; 5 CNRS, Aix Marseille Univ, FR3098, ECCOREV, F-13545 Aix-en-Provence, France; ilja.reiter@cnrs.fr

**Keywords:** climate change, drought, forest restoration, microbiome-assisted forest restoration, plant–bacteria interactions, synthetic communities, tree seedlings, extracellular polymeric substances, biofilm

## Abstract

Climate-change-induced drought threatens forest restoration by limiting seedling establishment. To address this, we developed synthetic bacterial communities (SynComs) tailored to support drought tolerance in two Mediterranean tree species, *Quercus pubescens* and *Sorbus domestica*. Bacteria were isolated from forest soil exposed to long-term drought, sampling across soil depths and root-associated compartments. We selected strains with key plant-beneficial traits, including exopolysaccharide (EPS) production, hormone synthesis (auxin, ABA), siderophore release, and osmotic tolerance. SynComs were assembled based on functional complementarity and ecological origin. Biofilm assays showed that even weak individual producers could enhance community-level performance. After initial screening on *Arabidopsis thaliana*, the most and least effective SynComs were tested on *Q. pubescens* and *S. domestica* seedlings. Compared to controls, the best-performing SynComs reduced the proportion of drought-symptomatic seedlings by 47% in *Q. pubescens* and 71% in *S. domestica*, outperforming single-strain inoculants. Notably, EPS-rich SynCom B aligned with the conservative root traits of *Q. pubescens*, while hormone-rich SynCom F matched the acquisitive strategy of *S. domestica*. Predictive modeling identified bacterial identity and symptom timing as key predictors of drought resilience. Our results highlight the value of matching microbial traits with plant strategies and drought context for climate-smart forest restoration.

## 1. Introduction

Climate change is profoundly disrupting ecosystems, particularly forests, through rising temperatures and altered precipitation patterns linked to greenhouse gas emissions [1]. These shifts are intensifying extreme weather events, including droughts and floods, which increasingly threaten forest stability and health [2,3].

Forest restoration remains a key strategy for climate change mitigation and ecological recovery, especially when grounded in the use of native species and community engagement [4]. Yet, its effectiveness is challenged by climate extremes that impair seedling survival during establishment. This growing vulnerability calls for expanded nature-based solutions to improve resilience in forest restoration programs [5,6].

Plants employ complex strategies to recruit beneficial microbiomes involving chemical signals, immune regulation, and microbial selection [7]. Under drought, they release specific root metabolites that signal stress and selectively attract plant-associated microorganisms [8], thereby enhancing resilience [9,10]. These interactions highlight secondary metabolites as central agents in coordinating plant–microbe–environment communication [11].

Plant-associated microbes (PAMs), such as growth-promoting rhizobacteria (PGPB) and mycorrhizal fungi, are widely recognized for enhancing drought tolerance in crops by improving nutrient uptake, hormone balance, osmotic regulation, and stress protection [12]. However, their application in forest trees remains underexplored. Promising results have been reported: Tiepo et al. [13,14] demonstrated the efficacy of commercial PGPB strains in boosting drought tolerance in Neotropical tree seedlings, while Khosravi et al. [15] identified native, drought-tolerant microbial strains that significantly improved oak seedling performance under drought.

Despite this, forest research has largely focused on broad soil processes, often overlooking specific microbe–tree interactions [16]. While mycorrhizal symbioses are well-established in both crops and trees [17], the role of bacteria differs. In forests, emphasis is placed on mycorrhization helper bacteria—especially phosphate-solubilizing strains—to support symbiotic networks [18], whereas agriculture prioritizes PGPB to counteract soil degradation. This contrast underscores the need for more targeted studies on bacterial support for tree seedlings facing drought.

Synthetic microbial communities (SynComs) generally outperform single-strain inoculations in helping crops face drought stress [19]. Recent studies highlight diverse strategies based on the bottom–up or top–down strategies for constructing effective SynComs, emphasizing ecological relevance, functional redundancy, and stability [20,21]. However, challenges like microbial interactions and environmental variability add additional layers of complexity, making it clear that SynCom development is highly context-dependent and lacks a one-size-fits-all solution.

Belowground carbon allocation plays a key role in soil processes, with roots and mycorrhizal fungi driving carbon and nutrient cycling. Root strategies are traditionally classified as “fast” (thin, short-lived, high nutrient uptake) or “slow” (thicker, longer-lived, adapted to low-resource conditions) [22]. Recent insights introduce a second axis centered on fungal collaboration: plants either invest in high specific root length to forage independently or “outsource” resource acquisition through symbiotic networks, forming thicker roots in the process [23]. This expanded “root economics space” integrates both root construction and fungal and bacterial symbiotic reliance, offering a more nuanced view of the belowground strategy [24]. Drought can further shift these strategies by altering root thickness [25] and reducing nutrient mobility, thereby increasing the carbon investment required for root and mycorrhizal structures [26]. Understanding how these strategies interact with SynComs offers promising insights for enhancing drought tolerance.

**Figure 1:**
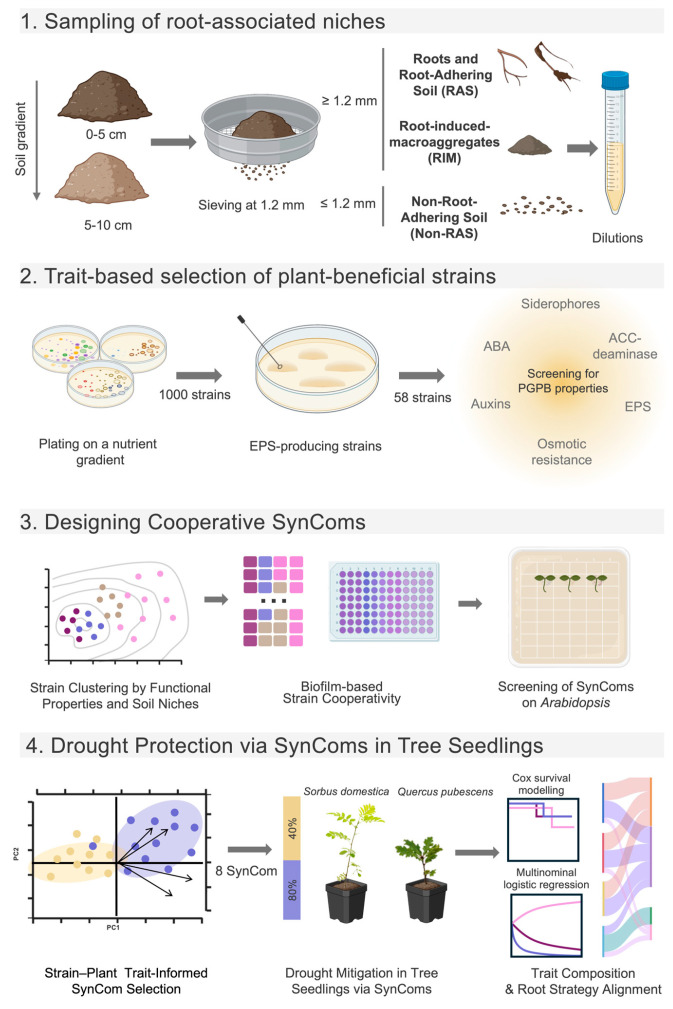
Workflow for designing and evaluating drought-resilient synthetic microbial communities (SynComs). Soil with a drought legacy (partial rain exclusion) was sampled at two depths (0–5 cm and 5–10 cm) and subdivided into four root compartments: Roots, Root-Adhering Soil (RAS), non-Root-Adhering Soil (non-RAS), and Root-Induced Macroaggregates (RIM). Bacterial isolates were screened for key plant growth–promoting traits (exopolysaccharide, auxins and abscisic acid production, ACC deaminase, siderophores, osmotic tolerance) and grouped via multiple correspondence analysis (MCA). Four-strain SynComs were assembled to maximize soil spatial and functional complementarity and tested for biofilm-based synergy. Top-, lowest- and mid-performing SynComs, identified from a Principal Component Analysis (PCA) on biofilm synergy and *Arabidopsis thaliana* assays, were further evaluated on *Quercus pubescens* and *Sorbus domestica* seedlings under drought and well-watered (40% and 80% field capacity) conditions. Survival modeling (Cox and multinomial logistic regression) assessed SynCom’s performance and key predictors of resilience. (Created with BioRender).

In this context, our objective was to develop nature-based solutions by designing tailored bacterial consortia (SynComs) inspired by soil niches and gradients, aiming to enhance drought tolerance in tree seedlings during their vulnerable establishment phase (Figure 1). We hypothesized that SynCom’s success would depend on the alignment between microbial traits and host plant strategies.

To capture trait diversity shaped by long-term drought, we isolated bacterial strains from a Mediterranean forest soil subjected to a 10-year partial rain exclusion. Sampling was performed across two depths and four root-associated compartments—Roots, Root-Adhering Soil, non-Root-Adhering Soil, and Root-Induced Macroaggregates. We prioritized strains producing exopolysaccharides, known to improve water retention, and selected complementary plant-growth-promoting traits such as abiotic stress tolerance, hormone production, and nutrient solubilization.

Using a trait-driven approach, we then assembled four-strain SynComs combining complementary functions reflective of forest soil heterogeneity. These were evaluated for cooperative biofilm formation, a proxy for interspecific ecological interactions [27], and for their plant-growth-promoting effects on *Arabidopsis thaliana*. Multivariate analyses helped identify high-performing and ineffective SynComs to validate our screening strategy.

To test the ecological relevance of our strategy, we inoculated selected SynComs on two Mediterranean tree species, *Q. pubescens* and *S. domestica*, with contrasting root traits. Our findings demonstrate that SynCom’s performance depends not only on microbial functional traits but also on their ecological origin and alignment with host plant strategies, highlighting the importance of designing microbial consortia that are both functionally and ecologically compatible with their target environment.

## 2. Results

### 2.1. Bacterial Strain Isolation

Soil was collected from the O3HP experimental site (Oak Observatory at the Observatoire de Haute Provence–OHP), a downy oak (*Quercus pubescens*) forest in southern France. Since 2012, this site has been equipped with a partial rain exclusion system that simulates a 35% reduction in spring and summer rainfall, creating intensified drought conditions for studying ecosystem responses to climate change [28].

We collected samples from both 0–5 cm (litter removed) and 5–10 cm depths of the A horizon. These layers differ in physicochemical properties, such as organic matter, pH, and calcium carbonate content [29], influencing microbial communities. We further subdivided the soil into four compartments based on proximity and interaction with plant roots: (1) the Root itself, which harbors specialized mutualists and endophytes [30,31], (2) Root-Adhering Soil (RAS), enriched in metabolically diverse genera such as *Pseudomonas*, *Bacillus*, and *Streptomyces* [30], (3) Root-Induced Macroaggregates (RIM), supporting oligotrophic, fungal-associated taxa [31,32], and (4) non-Root-Adhering Soil, acting as a reservoir of microbial diversity [31].

**Figure 2:**
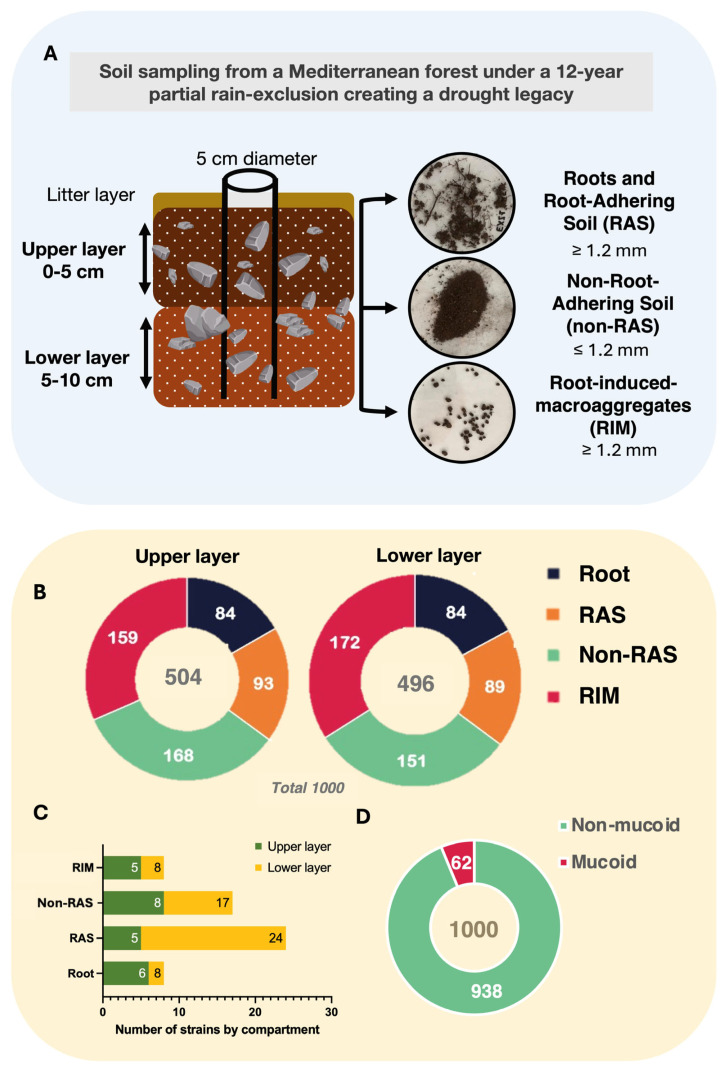
(**A**) Schematic diagram of sampling from soil submitted to an amplified drought. (**B**) Number of bacterial strains isolated at two soil depths, 0–5 cm and 5–10 cm. We partitioned the soil niche into distinct compartments surrounding the root: the root itself, the Root-Adhering Soil (RAS), the Root-Induced Macroaggregates (RIM), and the non-Root-Adhering Soil. (**C**) Distribution of bacterial isolates across soil depths and compartments; (**D**) Number of mucoid strains isolated from each compartment. Isolation of strains on 10-, 20-, and 50-fold diluted tryptic soy broth agar (TSA).

A total of 1000 microbial strains were isolated under different culture medium nutrient availability (Tryptic Soy Agar (TSA) diluted 10-, 20-, and 50-fold) from soil samples across two depth layers (0–5 cm and 5–10 cm), and four root-related compartments (Roots, RAS, non-RAS, RIM), to capture a wide diversity of growth strategies (Figure 2). From these, 504 isolates were obtained from the upper and 496 from the lower soil layer. In both layers, non-RAS and RIM accounted for the majority of isolates, with smaller contributions from RAS and Roots (Figure 2B,C).

Among the 1000 strains, 58 (5.8%) showed a strong mucoid phenotype (Figure 2D) and were screened for PGPB core traits (Supplementary File S1), including phytohormone production, nutrient solubilization, and stress tolerance [33]. Auxin (indole-3-acetic acid, IAA) production was strictly tryptophan-dependent, with 18 strains producing ranging from 0.84 to 58.92 µg mL^−1^. Additionally, 41 isolates (71%) exhibited ACC-deaminase activity, and all mucoid strains grew at −0.51 MPa, indicating high osmotic stress tolerance. Fitness assays identified 54 strains (93%) of interest, including 51 tolerant, 3 resistant (Figure S1), and 28 (48%) producing hydroxamate-type siderophores. Of these, 12 were pyoverdine-producing, typically linked to *Pseudomonas* under iron-limiting conditions.

**Figure 3:**
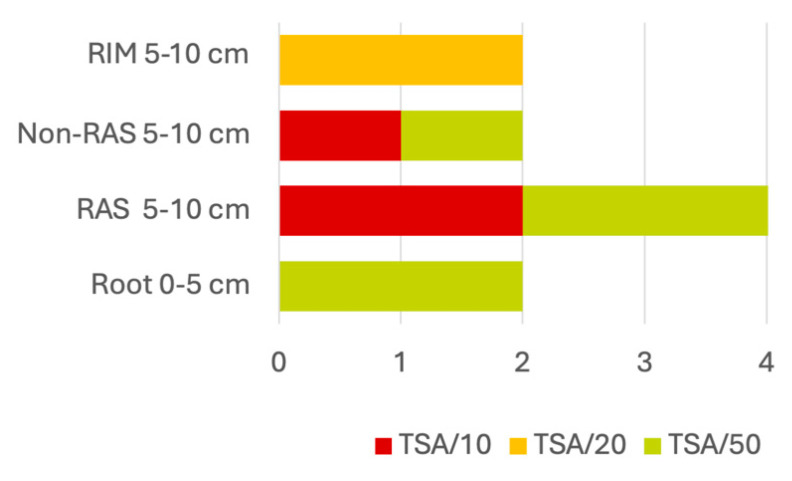
Twelve top-performing isolates. Distribution and numbers across soil depths, root-associated compartments, and culture medium. The soil was sampled at two depths (0–5 cm and 5–10 cm) and divided into four compartments: Roots, Root-Adhering Soil (RAS), non-Root-Adhering Soil (non-RAS), and Root-Induced Macroaggregates (RIM). Strains were grown on tryptic soy agar (TSA) diluted 10×, 20×, or 50× to capture different metabolic strategies. Bars (or sections) show the number of isolates obtained from each depth–compartment–medium combination.

Based on their overall traits, twelve top-performing isolates were selected to represent the spatial and nutritional diversity of the root–soil ecosystem (Figure 3). These included strains from four genera—*Peribacillus*, *Pantoea*, *Pseudomonas*, and *Caballeronia*—identified through full-length 16S rRNA gene sequencing (Supplementary File S1). Two isolates were identified as *Peribacillus simplex* (s1 and s57), one as *Pantoea pleuroti* (s14), and one as *Caballeronia glathei* (s48). Notably, the genus *Pseudomonas* was dominant, comprising multiple species, including *P. umsongensis* (s28), *P. migulae* (s31), *P. silesiensis* (s39 and s42), *P. mandelii* (s40 and s54), *P. lini* (s59), and *Pseudomonas* sp. (s41).

The isolates were further evaluated for plant growth-promoting properties, including salt tolerance, exopolysaccharide (EPS) production, and abscisic acid (ABA) synthesis. Most strains tolerated 0.5 M NaCl, with *P. pleuroti* s14 showing halotolerance up to 1.5 M NaCl. ABA production was detected only in *P. umsongensis* s28 (0.39 µgL^−1^ at 0.5 M NaCl), while EPS was produced by four strains, including *P. simplex* s12 and s57, *C. glathei* s48, and *P. lini* s59 (Supplementary File S1).

Growth dynamics were also assessed using two key parameters: t_mid (time to reach half of carrying capacity) and t_gen (doubling time), summarized in Figure S2. Nine strains—including s12, s14, s28, s31, and s39—exhibited fast growth (t_gen < 1 h and t_mid < 9 h), while three others (s41, s48, s49) displayed slower growth with doubling times over 1.5 h and t_mid above 10 h.

### 2.2. Multivariate Analysis of Strain Clustering Based on Functional Traits, Spatial and Nutrient Gradients

**Figure 4:**
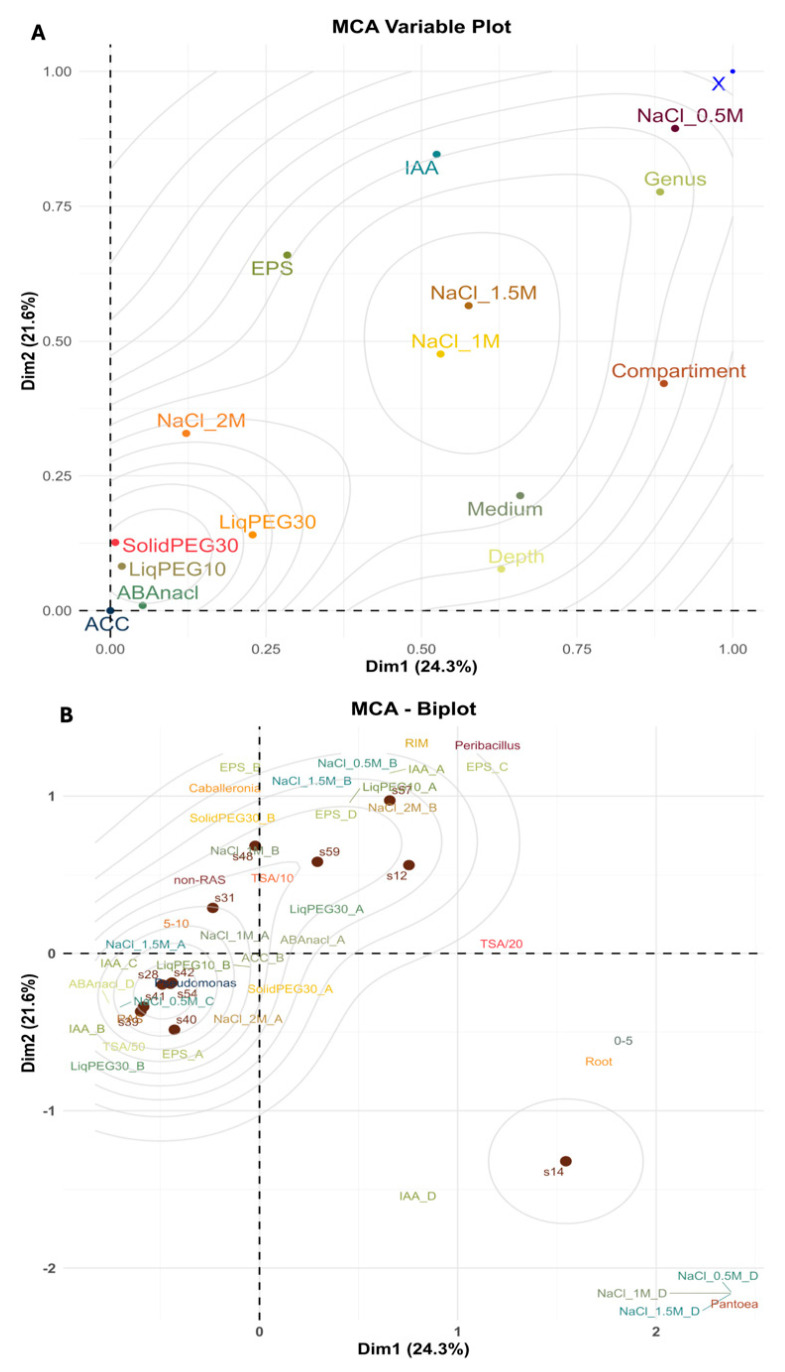
Multiple correspondence analysis (MCA). (**A**) MCA Variable plot showing the contribution of ecological and functional variables (centroids) to the first two dimensions, Dim 1 and Dim 2. Ecological variables are as follows: soil compartment (Roots; Root-Adhering Soil, RAS; non-Root-Adhering Soil, non-RAS); and Root-Induced Macroaggregates, RIM) and depth (0–5 cm and 5–10 cm), culture medium (Tryptic Soil Agar, TSA diluted 10-, 20-, and 50-fold). Functional variables are exopolysaccharide production (EPS), osmotic tolerance to polyethylene glycol (solid or liquid culture assays, PEG 1 0%), and to sodium chloride (NaCl 0.5, 0.1 and 1.5 M), auxin (IAA) and abscisic acid (ABA) production, ACC-deaminase activity (ACC), and strain genus. (**B**) MCA biplot shows the relationships between individual strains (brown dots) and the ecological and functional variables. A, B, C, and D letters indicate expression levels: A = low or none, B = moderate, C = high, and D = very high. Lines represent the iso-response values for the variable indicated.

We conducted a multiple correspondence analysis (MCA) to explore associations between bacterial strains and their plant growth-promoting traits across spatial and nutrient gradients (Figure 4A). Functional numerical variables (EPS production, IAA and ABA production, ACC-deaminase activity, and osmotic/halotolerance) were converted into ordinal categories ranging from A to D prior to MCA to enable correspondence-based analysis. Category A corresponded to low or no expression, B to moderate expression, C to high expression, and D to very high expression.

The first two dimensions explained 45.9% of the total variance. Separation along Dimension 1 (Dim 1) was primarily driven by ecological factors—namely compartment, isolation medium, and sampling depth—which were strongly associated together and contributed substantially to this axis structure. Dimension 2 (21.6%) captured variation in functional traits, particularly high EPS production, IAA production, and strong osmotic and halotolerance (NaCl 2 M). Interestingly, lower osmotic and halotolerance (NaCl 0.5–1.5 M) and genus-level taxonomic affiliation were highly correlated and contributed almost equally to both dimensions. In contrast, PEG-osmotolerance, ACC deaminase activity, and ABA production showed limited discriminative power in this analysis and were not strongly associated with either axis.

The MCA biplot displays the distribution of bacterial strains in relation to their categorical traits (Figure 4B). The analysis revealed four distinct strain clusters along the two main dimensions (Figure 4B). One cluster, represented by strain s14, appeared isolated on the far right of Dimension 1 and the lower end of Dimension 2. The remaining strains formed a gradient from the upper right to the lower left quadrant, reflecting mixed ecological and functional traits. Based on this distribution, four strain classes were defined: (i) s14—high IAA production and strong osmotic tolerance, isolated from 0–5 cm Roots; (ii) s12 and s57—high EPS producers with strong osmotic tolerance, from 0–5 cm Roots and 5–10 cm RIM, respectively; (iii) s31, s48, s59—strong EPS and moderate IAA production with intermediate osmotic tolerance, from 5–10 cm RAS, non-RAS, and RIM; (iv) s28, s39, s40, s41, s42, s54—low osmotic and halotolerance, with intermediate IAA, mostly from 5–10 cm RAS or non-RAS compartments.

### 2.3. Designing Synthetic Communities (SynComs)

Recent studies have shown that microbial consortia often exhibit enhanced functional capabilities compared to single-strain inoculants, particularly under complex environmental conditions [34,35]. We applied a consortium-based approach using four-strain combinations selected according to the spatial origin and nutrient context of their isolation sites. From an initial pool of twelve strains, 495 theoretical four-strain combinations were possible.

Based on the MCA biplot and the resulting trait-based clustering into four classes, we focused on strains isolated from 0–5 cm Roots and associated with strong functional potential—specifically those including either *P. pleuroti* s14 (Class 1), *P. simplex* s12 (Class 2), or both. We first generated 36 combinations by starting with s14 (Class 1), then adding one strain from Class 2, one from Class 3, and one from Class 4. This process was repeated twice: once excluding s12 (18 combinations) and once starting with s12 and excluding s14 (18 combinations), resulting in a total of 72 selected SynComs (Supplementary File S2).

### 2.4. Biofilm Assay

We evaluated synergistic/antagonistic biofilm production [36,37] among 12 bacterial strains arranged into 72 four-strain consortia (Table S1). Relative to their best-performing single strains in the consortium, the screening revealed synergistic interactions enhancing biofilm production in 8 consortia and negative interactions reducing biofilm formation in 10 consortia (Figure S3). The remaining consortia showed no significant change in biofilm production.

### 2.5. In Vitro Inoculation of Arabidopsis with Single Bacterial Strains or Consortia

We assessed the plant-growth-promoting potential of 12 individual bacterial strains and eight consortia exhibiting contrasting biofilm formation, categorized as highest (A: 12-48-59-41; B: 12-48-59-54; C: 14-57-59-54; D: 14-57-48-28), lowest (E: 14-12-31-42; F: 12-31-48-28), and intermediate (G: 12-48-59-39; H: 14-48-59-41) biofilm producers. To improve readability, the strain prefix “s” (e.g., s1–s12) was omitted when listing strains within SynCom compositions.

Seedlings were grouped into three developmental categories based on observed growth patterns: (i) germination, (ii) growth (continued development after germination), and (iii) advanced development, characterized by large rosettes (diameter ≥ 0.7 cm), at least six leaves, and more than ten lateral roots. Germination rates remained consistently high (>90%) across all treatments, including controls, with no significant enhancement observed from any consortium or individual strain (Figures S4–S6). Inoculation, whether with consortia or single strains, did not significantly influence seedling development (Figures S4–S6), regardless of biofilm interactions. Two exceptions were strain 41 (*Pseudomonas* sp.), which markedly improved seedling establishment, and strain 54 (*P. mandelii*), which completely inhibited the emergence of well-developed seedlings (Figure S5). Although both strains exhibited similar in vitro traits—intermediate IAA production, osmotic tolerance, and biofilm formation—they produced contrasting effects on *Arabidopsis* development.

### 2.6. Data-Driven Selection of Top-Performing SynCom Candidates

**Figure 5:**
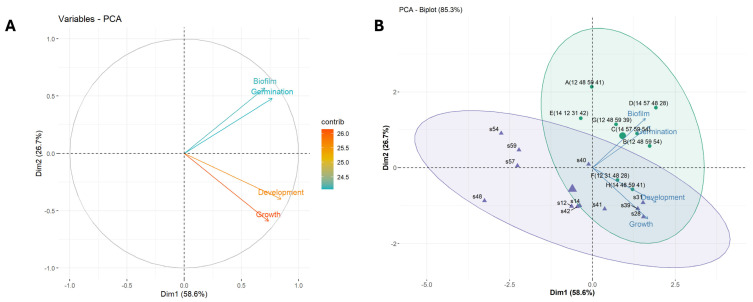
Principal Components Analysis of the biofilm assay and in vitro inoculation of *Arabidopsis thaliana* with single strains s1 to s12 or four-strain synthetic communities (SynCom) A to F. Variables are: Germination (percentage of germinated seeds), Growth (percentage of seedlings that continued growing after germination), Development (percentage of well-developed seedlings—seedlings with > 10 lateral roots and with rosettes ≥ 0.7 cm and at least six leaves). Ellipses 95% confidence interval. (**A**) PCA variable plot. Arrow direction shows correlation; color indicates variable contribution. (**B**) PCA biplot for individuals and variables.

To streamline the selection of the most effective single-strain inoculants and SynComs for young tree experiments, we performed a Principal Component Analysis (PCA) using four quantitative variables related to plant development: germination, growth, development, and biofilm formation. The goal was to reduce dimensionality and identify key associative features. The first two principal components explained 82.9% of the total variance, reflecting strong dimensional reduction and a reliable summary of the data. PC1 accounted for the majority of the variance (61.6%), with PC2 contributing an additional 21.3% (Figure 5A). PCA identified two distinct axes of variation, distinguishing biofilm formation and early-stage traits such as germination from later seedling responses, including growth and development. Growth and development in *A. thaliana* were positively correlated and loaded primarily on PC1, while biofilm production and germination were positively correlated and clustered along PC2. The orthogonality of these vectors indicates that early and late plant responses varied independently across treatments. Contribution values were relatively balanced (as shown by the color scale), indicating that all four variables contributed meaningfully to the PCA dimensions.

The PCA biplot (Figure 5B) revealed two distinct clusters, highlighted by green and violet ellipses. The first cluster grouped SynComs that were positively associated with biofilm formation and plant growth traits—either germination (A, C, and D), growth and development (F and H), or both (B). The second cluster consisted mainly of single strains that showed weak or negative associations with the measured traits, except for s28, s31, and s39, which were strongly linked to growth and development.

Based on this analysis, SynComs B, F, and H were identified as the best performers for promoting plant growth and development, whereas SynComs A and D were among the least effective. Among the top-performing individual strains, s31 and s39 were selected over s28 due to their superior tolerance to osmotic stress (Supplementary File S1).

### 2.7. SynComs and Single Inoculants to Mitigate Drought Stress in Tree Seedlings

Five weeks after inoculation for *Q. pubescens* and four for *S. domestica*, watering was withheld until soil moisture reached 40% field capacity (FC), which was then maintained. Over the following five weeks, plants were monitored for drought symptoms such as wilting leaves, discoloration, or premature leaf drop (Figure S7). The percentage of plants that remained symptom-free served as a proxy for drought tolerance, reflecting their capacity to maintain normal physiological processes despite reduced water availability (https://foresteurope.org/drought-adaptation-of-forests-in-europe-practical-strategies/, accessed on 10 April 2025).

Kaplan–Meier survival curves (KM curves) and log-rank test (LRT) were used to assess differences between the groups. The hazard function plays a very important role in survival analysis. The Cox proportional hazards model (Hazard Ratio, HR; Wald’s test) investigates the relationship between predictors and the time-to-event through the hazard function.

Survival analysis of *Q. pubescens* seedlings subjected to drought conditions (Figure S7a,b) revealed significant differences among bacterial treatments (KM curves, LRT, *p*-value = 0.0052). Consortium B provided the strongest protective effect with an HR of 0.53 (95% CI [0.30–0.97], *p*-value = 0.039), indicating that the risk of drought symptoms was reduced by approximately 47% compared to the water control. Single-strain inoculations did not significantly improve drought tolerance in QP seedlings relative to the controls (log-rank test, *p*-value = 0.12).

In *S. domestica* seedlings (Figure S7c,d), survival analysis also showed significant differences across bacterial treatments (log-rank test, *p*-value = 0.017). Consortium F emerged as the most effective treatment with an HR of 0.29 (95% CI [0.11–0.77], *p* = 0.013), indicating a 71% reduction in risk compared to water. Pairwise comparisons confirmed that Consortium F significantly outperformed both the water control (pairwise log-rank test, *p*-value = 0.030) and its constituent strains S31 and S39 (pairwise log-rank test, *p*-value = 0.004 and 0.006, respectively). Strains S31, S39, and S48 alone did not significantly differ from the water control, with HR *p*-values all above 0.05. This suggests that the enhanced drought protection observed in *S. domestica* seedlings inoculated with Consortium F likely resulted from synergistic interactions among its component strains. These results contrast with those for *Q. pubescens*, highlighting species-specific responses to bacterial inoculation. No other consortia tested showed improved survival compared to water or TSB/100 controls (LRT, *p*-value = 0.48).

### 2.8. Prediction of Inoculant Efficacy for Drought Protection of Quercus and Sorbus Seedlings

Multinomial logistic regression model (MLM) is used to predict the relationship between one nominal dependent variable (with more than two categories) and one or more independent variables, which can be either categorical or continuous (https://resources.nu.edu/statsresources/Multinomiallogistic, accessed on 10 April 2025). We used MLM to model the relationship between the outcome of drought symptoms on trees, using the kinetics of symptoms, treatment type, and increments in stem height and diameter.

### 2.9. Quercus pubescens

In *Q. pubescens* experiments with consortia A, B, D, and H (Figure S8A), the models showed that symptom kinetics and stem diameter increment were the most crucial factors in predicting the outcome of the inoculation of A, B, and D (A, χ^2^ = 486, df = 16, *p*-value < 0.001; B, χ^2^ = 418.97, *p*-value < 0.001; D, χ^2^ = 42.53, *p*-value < 0.001). Height increment showed no significant impact (χ^2^ = 2.192, *p*-value = 0.335). Treatment with Consortium D significantly reduced the occurrence of partially affected phenotypes from 24.7% (±3.5%) to 14.2% (±3.1%) (*p*-value = 0.033) but showed no significant effect on severe symptoms.

When modeling the inoculation with single strains and Consortium F (Figure S8B), neither diameter increment (χ^2^ = 2.182, *p*-value = 0.336) nor height (χ^2^ = 0.0524, *p*-value = 0.974) was significant according to the likelihood ratio test. Only time remained highly significant (χ^2^ = 143.277, *p* < 0.001), with treatment effects showing modest impact (χ^2^ = 20.741, *p*-value = 0.023). This suggests that growth parameters are not predictive variables in the case of moderately effective bacterial treatments, while temporal progression of symptoms remains the dominant factor.

### 2.10. Sorbus domestica

In *S. domestica* treated with consortia A, B, D, and H (Figure S8C), the MLM showed that diameter increment, height increment, treatment, and time significantly predicted symptom development (χ^2^ = 146, df = 21, *p* < 0.001), though with a moderate 11.6% explanatory power (R^2^N = 0.116). When treated with consortia F and single strains S31, S39, and s48 (Figure S8D), the predictor variables stayed significant, although with a lower fit (χ^2^ = 154, df = 21, *p* < 0.001, R^2^N = 0.121).

The model predicted that consortia A (*p*-value = 0.001) and D (*p*-value = 0.04) impacted the development of the partially affected symptoms, and consortia F (*p*-value = 0.008) impacted the development of very affected symptoms. The predicted probability of developing partial symptoms under drought conditions was 24.2 ± 3.5% in the water control group, which decreased to 12 ± 3% with consortium D inoculation and further declined to 3 ± 1.6% with consortium A inoculation. The inoculation with consortium F decreased the probability of the development of very-affected symptoms, from 14.1 ± 2.8% to 4 ± 1.8%.

## 3. Discussion

This study investigated nature-based solutions using tailored bacterial consortia (SynComs) derived from Mediterranean forest soils to enhance drought tolerance in tree seedlings, particularly during the critical establishment phase. We used soil that has been subjected to recurrent amplified drought conditions since 2012, through the exclusion of spring and summer rainfalls, reducing annual rainfall by approximately 35%—mimicking the climate projections for 2050 and 2100 for RCP 8.5 and 4.5, respectively [38]. The concept of using bacterial inoculants that have already experienced drought is rooted in the ‘drought legacy effect’ [39]. Through repeated exposure to water deficit, these bacterial communities potentially develop or refine mechanisms for drought tolerance and effective host interaction in harsh environments) [40].

### 3.1. From Soil Niches to Synthetic Communities (SynCom)

Bacterial communities associated with plant roots display distinct compositional and functional traits across soil compartments, shaped by gradients in plant influence, microbial interactions, and environmental conditions. By sampling multiple compartments surrounding the root system—Roots, Root-Adhering Soil (RAS), non-Root-Adhering Soil (non-RAS), and Root-Induced Macroaggregates (RIM)—at two depths (topsoil: 0–5 cm, without litter, and subsoil: 5–10 cm), and culturing on a carbon-gradient medium (serial dilutions of Tryptic soil agar, TSA), we isolated bacterial strains spanning a range of metabolic strategies and nutrient requirements. Although both soil layers yielded an equal number of isolates, the subsoil (5–10 cm)—which retains significantly more moisture under drought (water content at 10 cm depth was 100 times higher than at the surface)—provided the majority (84%) of high-performing strains. This depth is known to promote deeper rooting and greater exudation [41], suggesting that it represents a particularly productive niche for isolating drought-alleviating, plant-growth-promoting bacteria (PGPBs).

The compartment Roots yielded *Peribacillus simplex* and *Pantoea pleuroti*, both recovered from the richest culture medium used in this study (TSA/10), highlighting their adaptation to carbon-rich microenvironments at the root interface. *Peribacillus simplex* is a versatile bacterium primarily isolated from soil and crop rhizosphere—where it exhibits plant-growth-promoting and biocontrol properties. *P. simplex* is also found in harsh environments such as arid soils and stratospheric air samples (reviewed in [42]). *P. pleuroti*, associated with *Pleurotus* mushroom fruiting bodies, is mainly saprotrophic and occasionally pathogenic to its fungal host [43]. These isolates reflect differing ecological niches and carbon usage profiles, with *P. simplex* demonstrating broader metabolic flexibility [44] while *P. pleuroti* requirements are inferred from related species [45].

In RAS, *Pseudomonas* spp. dominated the culturable bacterial community [46]. Several close relatives from our *Pseudomonas* collection have been previously isolated from carbon-poor or recalcitrant niches. *P. migulae* was recovered from mineral water [47] and from the endophytic root tissues of lodgepole pine (*Pinus contorta*) growing in unreclaimed mining sites in British Columbia [48]. Similarly, *P. mandelii* and *P. lini* were isolated from the stem and needles, respectively, of the same pine species [48]. These findings highlight the niche-specific adaptations of *Pseudomonas* strains, while shared traits—such as exopolymer production (this study) and the ability to degrade complex substrates—underscore the remarkable metabolic diversity of this bacterial genus.

From RIM, we isolated *P. lini* and *P. simplex*, both confirmed strong exopolysaccharide (EPS) producers. EPS stabilizes macroaggregates and supports biofilm formation [49]. These EPS-producing strains contribute to rhizosheath formation and drought tolerance.

In non-RAS, *Caballeronia glathei* and *P. mandelii* were isolated. *C. glathei* (formerly *Burkholderia* [50]) is commonly isolated from bulk soil, rhizosphere soil, and contaminated environments such as wastewater sludge. *C. glathei*, isolated from acid lateritic relicts in Germany [51], is metabolically versatile, nitrogen-fixing, and tolerant of contaminants [52]. *P. mandelii* exhibits traits such as polyhydroxybutyrate accumulation under nutrient stress [53], aiding survival and pollutant degradation. This trait is considered a survival strategy, enabling the bacterium to store excess carbon during nutrient scarcity and contributing to the degradation of environmental pollutants [53].

Although EPS supports plant growth by enhancing moisture retention, ion immobilization, and microbial colonization, only 5.8% of our isolates formed mucoid colonies—a common proxy for EPS production. EPS synthesis is typically triggered by osmotic stress and a high carbon-to-nitrogen (C/N) ratio [54]. EPS-producing bacteria contribute to the creation of microenvironments that retain water, immobilize harmful ions, and promote a healthier rhizosphere [55,56]. They also facilitate soil aggregation by interacting with clay particles, thus helping to maintain the soil’s mechanical and physical properties [57,58]. Santaella et al. [59] demonstrated that *Rhizobium* sp. YAS34, an EPS-producing strain, promoted the formation of stable microaggregates composed of EPS, mineral particles, and organic matter. These EPS-rich biofilms were mainly localized at the root base, lateral root emergence zones, and root hairs. In contrast, no EPS accumulation was observed at the root apex, where mucilage and root-cap-derived cells typically form the first interface with soil [60]. This tri-partite interaction between EPS, microorganisms, and the host plant enhances drought tolerance by improving soil structure, protecting roots, and mitigating environmental stress—ultimately supporting more efficient water uptake under dry conditions [56].

We also assessed classic PGPB traits, including auxin and ABA biosynthesis, siderophore production, ACC deaminase activity, and tryptophan dependency. IAA synthesis was strictly tryptophan-dependent, implying reliance on root exudates. Siderophores enhance iron acquisition and can trigger plant immune responses [61]. The interest in ABA-producing bacteria relies on their ability to signal drought to the plant host [62]. We identified *P. usmongensis* s28 as an ABA-producing bacteria (0.39 µgL^−1^ under 0.5 M NaCl) in the range of *Bacillus amyloliquefaciens* RWL-(0.32 ng mL^−1^) [62], *Bacillus pumilus* (45 pmol mL^−1^ [63]), and *Bradirhizobium japonicum* USDA 110 (0.019 µgmL^−1^ [64]).

Altogether, over 55% of our isolates displayed at least four key PGPB traits, and 8 of the final 12 strains retained 75% of nine tested functions.

The MCA revealed distinct ecological and functional structuring within the bacterial isolate pool, highlighting key factors that drive strain differentiation. Spatial variables—soil compartment, isolation medium, and sampling depth—were the major contributors to the first principal axis, emphasizing the role of niche-specific adaptation in shaping microbial communities. In contrast, functional traits like EPS production, IAA biosynthesis, and salt tolerance—known to support drought tolerance—were primarily associated with the second axis. The independence of these axes suggests that ecological origin and functional capacity represent distinct yet complementary dimensions of microbial variation.

This insight guided our SynCom design strategy. Rather than relying solely on individual trait performance, we prioritized strains that not only exhibited beneficial traits but also thrived in different soil compartments. By assembling four-strain SynComs reflecting the spatial structure of the rhizosphere, we aimed to reduce niche overlap and microbial competition, fostering functional complementarity and cooperation. Recognizing the limits of trait-based prediction [65], we used a multivariate selection approach to reduce the number of candidate SynComs while maintaining both ecological and functional diversity. While this pragmatic strategy proved effective, it may have excluded other relevant microbial traits—such as phenol oxidase activity [66], broad substrate metabolism [67], or additional plant-interactive capabilities [68].

In summary, combining spatially structured sampling, functional trait screening, and ecologically informed SynCom design [20] enabled us to identify bacterial consortia with strong potential to alleviate drought stress. However, context-dependent validation remains essential, as microbial performance cannot be fully predicted from traits or origin alone [65].

### 3.2. Stronger Together? How Weak Biofilm Formers Drive Collective Performance

Biofilm formation, a key adaptive trait under drought conditions, was used to associate cooperative strains. Although not the sole mechanism of plant growth promotion, biofilm formation enhances root colonization, microbial survival, and stress signaling, ultimately amplifying the effects of traditional PGPB traits such as nutrient solubilization and phytohormone production [69]. Biofilms serve as structured environments where cooperative metabolic exchanges can be intensified, although resource competition may favor dominant strains, potentially compromising community cohesion [70].

Among the 12 bacterial strains tested, seven—mostly from root-associated compartments—formed biofilms individually. *P. migulae* s31 and *P. mandelii* s40 were the most prolific producers as single strains. Paradoxically, some of the highest-performing consortia for collective biofilm production included strains that were weak individual producers (e.g., s12, s57, s59, s48). In contrast, strong individual producers such as s31 and s40 often suppressed biofilm formation in consortia, likely due to competitive dominance. This pattern illustrates a trade-off: strains optimized for individual performance may inhibit collective function, while weak biofilm formers may thrive in cooperative networks by benefiting from partner-derived EPS or metabolic scaffolding [71,72].

We selected twelve individual strains and eight representative SynComs for further testing on plants, grouped by their biofilm production levels: high (A, B, C, D), low (E and F), and intermediate (G and H). These were screened for their drought-alleviating and plant-growth-promoting capacities, allowing us to evaluate how biofilm-forming ability translates to functional performance in plants.

### 3.3. Teaming up Underground: Optimizing SynCom by Matching Microbial Skills to Root Strategies

Root strategies have traditionally been described along a fast–slow continuum: fast (acquisitive) roots are thin, short-lived, and specialized for rapid nutrient uptake, whereas slow (conservative) roots are thicker, longer-lived, and adapted to low-resource or stressful environments [22]. More recent frameworks propose a refined two-dimensional model—the “root economics space” (RES)—which integrates structural carbon investment and links root-associated microbial functions with root strategies [23,24,73,74]. Within this model, plants adopt two main strategies for acquiring soil resources: either through the development of long, fine roots with high specific root length—Do-It-Yourself (DIY) strategy—maximizing direct uptake, or by investing in thicker roots that support microbial symbioses and interactions, thereby outsourcing nutrient acquisition [23,75].

To explore these root strategies and their microbial partnerships, we selected *Arabidopsis thaliana* (*Brassicaceae*), *Quercus pubescens*, and *Sorbus domestica*. *Arabidopsis thaliana* is a non-mycorrhizal model plant [76] with fast, acquisitive root traits and genetic tractability [23]. The lack of fungal symbiosis places it at the “Do-It-Yourself” end of the RES gradient. *Quercus pubescens*, an ectomycorrhizal Mediterranean oak, exhibits a conservative root strategy featuring deep, woody roots and drought-adaptive traits like enhanced root tissue density and hydraulic conductance [77,78]. Under drought, it employs a multi-dimensional strategy that includes high root hydraulic conductance to maintain leaf water potential, coupled with moderately regulated stomatal conductance [78]. *Sorbus domestica* develops a shallow, highly ramified root system (https://images.wur.nl/digital/collection/coll13/id/1318/ (accessed on 10 April 2025); EUFORGEN; [79]) and adopts a drought-avoidance strategy by accelerating root growth and secondary root development, improving water capture across a larger soil area [80,81,82]. Both *Q. pubescens* and *S. domestica* are well-adapted to the Mediterranean climates and are considered future species for forest restoration in Europe under climate change.

Among tested strains, *P. silesiensis* s39 promoted growth in *A. thaliana*, whereas *P. mandelii* s54 inhibited primary root development—possibly due to cyanide production, which can suppress auxin signaling [83]. Nevertheless, under regular conditions, none of the SynComs significantly improved *A. thaliana*, suggesting its “DIY” strategy may limit benefits from microbial partnerships in non-stressful environments.

For *Q. pubescens* and *S. domestica* seedlings, a practical pre-plant soil inoculation method was used differing from the one used for *A. thaliana* and from Tiepo et al. [13,14] and Khosravi et al. [15]. Pre-plant soil inoculation before outplanting offers a practical advantage, as it can be conducted in the nursery and is more adaptable than direct seed or acorn inoculation. Under drought, two SynComs (F and B) significantly reduced stress symptoms in these tree species. SynCom F, associated with *A. thaliana* development, lowered the hazard ratio (HR) of drought symptoms by 71% in *S. domestica*, while SynCom B, linked to enhanced biofilm formation, seed germination, and *A. thaliana* vigor, reduced the HR by 47% in *Q. pubescens*. Critically, none of the single-strain treatments matched the efficacy of the full communities, emphasizing the importance of synergistic interactions [27].

Notably, 75% of strains in SynComs B and F were isolated from a medium with moderate carbon content (TSA/10) and 80% from the 5–10 cm depth, suggesting functional potential is not limited to more oligotrophic reservoirs or topsoil. Also, half of SynCom B and a quarter of SynCom F strains came from non-Root-Adhering Soil, including macroaggregates—a zone of intense microbial, fungal, and root interaction—highlighting overlooked microbial niches with cooperative potential.

Network analyses (Figure S8) revealed SynCom B to be a local optimum for *Q. pubescens*, where minor compositional changes diminished its performance. A key player was strain *P. mandelii* s54, detrimental in *A. thaliana* yet beneficial in *Q. pubescens*, highlighting the host dependency on microbial function. Likewise, SynCom F proved uniquely effective in *S. domestica* through the combination of s12, s31, s48, and s28. The ineffectiveness of s31 alone underscores the non-additive nature of SynCom’s performance.

**Figure 6:**
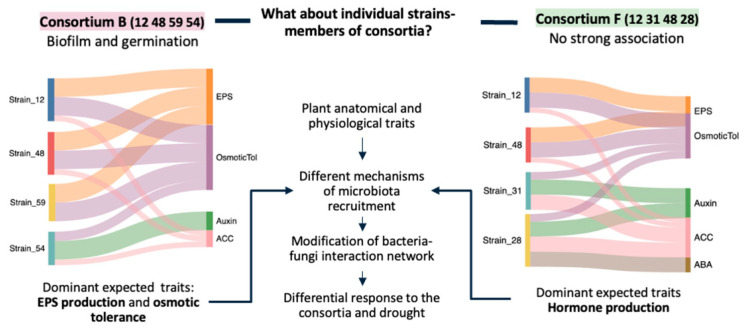
Alluvial diagram illustrating the compositions of the top-performing SynComs B (**left**) for *Quercus pubescens* and F (**right**) for *Sorbus domestica*. Flow widths represent the relative contribution of each strain’s key traits (e.g., exopolysaccharide (EPS) production, osmotic tolerance, hormone biosynthesis) within each optimized consortium.

Overall, SynCom efficacy appears to hinge on how well microbial functions align with a plant’s root strategy (Figure 6). In thin, fast-acquisitive roots like those of *S. domestica*, SynComs boosting nutrient uptake (e.g., iron solubilization or phytohormone production) is especially advantageous. For conservative, coarser roots, promoting “hyphal-like” biofilm networks or soil-structuring EPS may be more beneficial. These host-specific interactions suggest that tailoring SynCom design to each plant’s ecological and morphological traits is crucial for improving drought tolerance and resource use efficiency—especially in stress-prone conditions.

### 3.4. Beyond Survival: Predictive Insights into SynCom Function and Drought Tolerance in Trees

Our predictive modeling extended the survival analysis, offering deeper insights into SynCom’s impacts on drought symptom progression. SynCom D reduced the proportion of *Q. pubescens* seedlings with partial drought symptoms by 10.5% compared to the water control, while SynCom F significantly alleviated symptoms in *S. domestica*. Although models also predicted mitigating effects for SynComs A and D in oak, survival analysis showed only marginal changes in HR—29% for A (*p* = 0.051) and 6% for D (*p* = 0.725).

Bacterial single strain or SynCom treatment identity consistently emerged as a strong predictor, confirming its influence on symptom development. Time was also significant, reflecting drought symptom dynamics. In contrast, changes in plant height and diameter increments were not consistently predictive, suggesting that unmeasured anatomical or physiological traits may better explain tolerance patterns.

**Figure 7:**
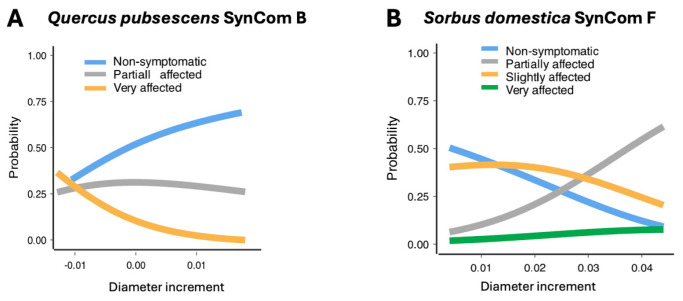
The probability of symptom development under the most efficient synthetic microbial communities (SynComs) for each tree species—calculated via estimated marginal means from a multinomial logistic regression model. (**A**) *Sorbus domestica* inoculated with SynCom F; (**B**) *Quercus pubescens* inoculated with SynCom B.

In *Q. pubescens*, a greater stem diameter increment was linked to a lower likelihood of severe symptoms, indicating better turgor or growth under drought (Figure 7A). This aligns with the literature showing that anatomical traits, such as xylem size and stomatal density, influence drought [84]. Stem diameter is a more sensitive and immediate physiological indicator of drought stress than height [85]. When the relative water content in plant tissues drops, the diameter decreases measurably due to tissue shrinkage, reflecting real-time water status. Further, drought reduces cambial activity and, thus, tree-ring increment. Additionally, the stem incorporates carbohydrate storage, which is reduced during growth. In contrast, plant height represents cumulative growth over time and cannot decrease under drought. The investigated species have determinant growth, such that the tree height would not change after establishment—unless a second flush would elongate these. Height is thus not responsive to short-term water deficits at this growth stage, eliminating it as a predictor of drought tolerance.

Interestingly, *S. domestica* showed the opposite pattern—greater stem diameter was associated with more partial symptoms (Figure 7B). As an anisohydric species with strong drought recovery [86], it may maintain growth during stress, increasing its risk of hydraulic failure. We observed that *S. domestica* often shed basal leaves, thereby reducing total leaf area while sustaining photosynthesis in upper canopy leaves, suggesting a resource allocation towards a higher use efficiency at the whole plant level.

Our findings reinforce that SynComs are generally more effective than single strains in improving drought tolerance. Stem diameter increment, particularly in inoculated seedlings, emerged as a promising trait linked to enhanced drought protection. These results suggest it could serve as a phenotypic marker for SynCom efficacy.

As George Box noted, “all models are wrong, but some are useful”. While informative, the model had modest explanatory power (R^2^_N = 0.121), which is inherent to MLR as compared to more constrained models and does not necessarily sign of poor model quality [87,88]. This suggests that other unmeasured variables may also contribute to the outcome.

Future work should leverage high-throughput phenotyping and multi-omics datasets—metagenomics, proteomics, metabolomics—combined with machine learning [20,89] to improve predictive power and guide SynCom design more effectively.

Overall, the effective SynComs can potentially be used to prevent *Q. pubescens* and *S. domestica* from experiencing drought stress after further investigation and field experiments.

## 4. Materials and Methods

### 4.1. Soil Sampling

The soil was sampled at the O3HP (Oak Observatory at the OHP) experimental facility, which is part of the European and French research infrastructure AnaEE-ERIC and AnaEE-France and is located at Saint-Michel-l’Observatoire in Alpes de Haute-Provence, France (43.9352° N, 5.710667° E). This facility runs a rain exclusion system since 2012 above 300 m^2^ of canopy to simulate an extended summer and an additional spring drought, cumulating to about 35% reduction in annual precipitation, as in line with predictions by climate models [1], in order to create an enhanced drought as compared to natural drought [38]. Exclusion of full rain events raises the number of dry days by about 30 per year [90]. The dominant tree species is *Quercus pubescens* (downy oak) mixed with co-dominant *Acer monspessulanum* L. (Montpellier maple), representing about 75% and 25% of the litter production and standing biomass, respectively. The soil is a calcosol (leptosol with a mollic horizon), consisting of two distinct horizons [29]: a mollic horizon (rich in organic matter, approximately 0–5 cm deep) and a leptosol horizon (bedrock with little organic matter, rich in clay, approximately 5–10 cm deep).

Groups of trees were used as focal points for subsequent soil sampling. Soil was collected using a stainless-steel core (5 cm diameter) from the rain exclusion zone on 22 October 2021. At the time of sampling, the soil had not received any direct rainfall since mid-June, and the volumetric soil moisture mean had been 0.136 L L^−1^ for 15 weeks since July 2021 and 13.34 L L^−1^ at 10 cm depth. The litter layer was discarded before coring, and samples were taken from 0–5 cm (upper layer) and 5–10 cm (lower layer) depths. Samples were transported to the laboratory in refrigerated boxes and immediately stored at 4 °C. Soils were sieved at 1.2 mm mesh to separate non-Root-Adhering Soil (non-RAS) from Roots (RT), Root-Adhering Soil (RAS), and Root-Induced Macroaggregates (RIM) (Figure 2B). Roots were vortexed in sterile water to separate the RAS.

### 4.2. Strain Isolation and Growth

A total of 1 g of soil or 100 mg of ground fresh roots was used to prepare the suspensions in 10 mL or 1 mL of sterile distilled water, respectively. The suspensions were homogenised by vortexing, and 100 μL were used for dilution plating on a non-selective Tryptic Soy Both (TSB, Soybean-Casein Digest Broth) diluted 10×, 20× and 50× times and solidified with 3 gL^−1^ of Agar (TSA). Plates were incubated at 30 °C for 48 h. Morphologically different isolated single colonies were picked up and re-streaked to fresh 10-fold diluted TSA plates and incubated in a similar manner. The purified cultures were stored in a 10-fold diluted TSA medium at 4 °C in a dark chamber.

Strain growth parameters in liquid 10-fold diluted TSB were measured using 96-well plates incubated at 30 °C and 3 mm of orbital amplitude (around 300 rpm) for 24 h. Optical density (OD) at 600 nm was recorded every 15 min using an Infinite^®^ M1000 plate reader (Tecan Group Ltd., Männedorf, Zurich, Switzerland). Two key growth parameters were extracted: the time to reach half of the carrying capacity (t_mid), which corresponds to the inflection point of the growth curve (Figure S2), and the minimum doubling time (t_gen), representing the fastest generation rate. Both parameters were calculated as indicators of growth performance (Allen et al., 2018 [91]).

### 4.3. Strain Screening for Specific Traits

Exopolysaccharide (EPS) production capacity was estimated by transferring the single colonies to 10-fold diluted TSA supplemented with 20 gL^−1^ of glucose, followed by incubation at 30 °C for 48 h in the dark. A quantitative estimate of exopolysaccharide production was made from colonies with a strong mucoid phenotype [59]. The dry mass was used to estimate the EPS production capacity.

Strains were screened for Plant-Promoting Growth Bacteria (PGPB) traits as siderophore production using casamino acid (CAS) and overlaid CAS (O-CAS) assays [92,93,94], plant hormone production, such as auxins [95,96]. Isolates with osmotic tolerance were screened at the osmotic potentials of −0.51, −1.23, and −2.56 MPa in the liquid phase and −0.64, −1.12, −2.28 MPa in the solid phase using polyethylene glycol (PEG 6000) [97]. Salt-tolerant strains were selected in liquid culture assays with NaCl 0.5, 1, 1.5, and 2 M [98].

### 4.4. Ability to Form Biofilms

To test for synergistic biofilm production among bacterial strains, we conducted in vitro assays using both single strains and four-strain consortia derived from various soil compartments. Biofilm formation was assessed using the 96-well peg lid system (MBEC Assay^®^ Biofilm Inoculator, Innovotec^®^, Edmonton, AB, Canada), incubated for 24 h at 30 °C without shaking, based on protocols from [36,37]. After incubation, biofilms were stained with crystal violet, washed, and the dye solubilized in ethanol for OD590 nm measurement using a microplate reader (Infinite^®^ M1000, Tecan, Palm Springs, CA, USA). Synergy was evaluated by comparing the OD590 of each consortium to the highest OD590 of its constituent strains. Ratios ≥ 1.9 indicated synergy; ratios < 0.7 suggested antagonism; intermediate values reflected minor differences. Thresholds were set empirically (Figure S9) to select a manageable number of consortia for further testing.

### 4.5. Strain Identification

DNA was obtained by heat shock of a single clone diluted in 20 µL sterile ultrapure water (Milli-Q MilliQ Millipore Ultrapure Water Purification System type I (Millipore, Merck KGaA, Darmstadt, Germany)). The full-length 16S rRNA gene was amplified using bacterial-specific primers 27F (5′-AGAGTTTGATCCTGGCTCG-3′) and 1492R (5′-GTTACCTTGTTACGACTT-3′). Amplification was performed using the GoTaq^®^ Flexi DNA Polymerase (Promega, Madison, WI, USA) under the following conditions: initial denaturation at 95 °C for 2 min followed by 34 cycles, each set at 95 °C for 30 s, 53 °C for 30 s, and 72 °C for 1:30 min, with a final elongation step at 72 °C for 5 min. Amplified PCR products were transferred to GENEWIZ (France) for two-way Sanger sequencing. The National Centre for Biotechnology Information (NCBI)’s Basic Local Alignment Search Tool (BLAST 2.13.0) software (http://blast.ncbi.nlm.nih.gov, accessed on 31 January 2023) algorithm was used to search for homology using an aligned contiguous consensus sequence of the 16S rRNA gene. The 16S rRNA gene sequences of the bacterial strains used in this study have been deposited in the NCBI GenBank database and are publicly available under the following accession numbers: https://submit.ncbi.nlm.nih.gov/subs/genbank/SUB15323576 (accessed on 31 January 2023) and https://submit.ncbi.nlm.nih.gov/subs/genbank/SUB15314851 (accessed on 31 January 2023).

### 4.6. Test of Single Strains and Consortia on Arabidopsis thaliana

*Arabidopsis thaliana* seeds (Col-0 ecotype) were sown in square Petri dishes (120 mm × 120 mm, Greiner) containing solid medium (two-fold diluted Hoagland with Phytagel^®^) and inoculated with 10 µL of either bacterial suspensions (10^6^ colony-forming units- CFU mL-1- calibrated from OD_600_ measurements) or sterile Milli-Q water, following Santaella et al. [59]. Plates were incubated in growth chambers (25 °C/20 °C day/night, 16 h photoperiod, PAR 100 µmol m⁻^2^ s^−1^, 40% relative humidity) for 3 weeks.

Germination was assessed on day 4, and root development was monitored every second weekday. Seedlings were categorized as germinated, developed, or well-developed (rosette > 0.7 cm, > 7 leaves, > 10 lateral roots). Additionally, the number and total length of first-order lateral roots per cm of primary root were recorded.

### 4.7. Greenhouse Experiments on Trees

Acorns of *Q. pubescens* Willd. (QPU751), collected in southeastern France (Pignans, Var), and seeds of *Sorbus domestica* (SDO900 FR), collected throughout mainland France, were provided by the ONF Sècherie de la Joux (France).

The experiment ran from January to November 2023 at the ONF-PNRGF (Pôle National des Ressources Génétiques Forestières) in Cadarache, France. The bacterial strains and consortia were tested in batches of six treatments on *Q. pubescens* and *S. domestica* in two separate trials, each batch including control treatments. A randomized complete block design was then employed to account for potential environmental heterogeneity within the greenhouse. Each block contained all treatments, including controls, enabling within-block comparisons and minimizing the effects of spatial variation (Figure S11).

Seven-month-old *Q. pubescens* and five-month-old *S. domestica* seedlings were grown in 1.4 L pots filled with potting soil at 80% field capacity (FC). This substrate consisted of equal parts (1:1 *v*/*v*) white peat and a granular organic fraction (Pine-bark-based organic material), with mineral fertiliser 12-12-14 (N–P_2_O_5_–K_2_O–MgO–SO_3_: 12–12–17–2–20, supplemented with Boron 0.02%, Iron 0.3%, and Zinc 0.01%.) additionally amended with 2 kg m^−3^ of Osmocote Exact 8/9 (ICL Growing Solutions, 15-9-11; N–P_2_O_5_–K_2_O–MgO: 15–9–11–2, enriched with trace elements: Boron 0.03%, Copper 0.050%, Iron 0.45%, including Iron-EDTA 0.09%, Manganese 0.06%, Molybdenum 0.020%, and Zinc 0.015%). The soil initially had a pH (H_2_O) of 6.2, which equilibrated to a pH (H_2_O) of 7.0 after five months. In August 2023, the seedlings were inoculated with a 100 mL suspension of either a single strain or a four-strain consortium. Bacterial cells were grown in a 10-fold diluted TSB medium and diluted 10-fold to achieve a final concentration of 10⁷ cells mL^−1^. MilliQ water and 100-fold diluted TSB (TSB/100) were used as controls (Figure S11).

Five weeks after inoculation for *Q. pubescens* and four weeks for *S. domestica*, half of the plants were subjected to drought, while the rest remained well-watered. Drought was induced by gradually reducing soil water content from 80% to 40 ± 5% field capacity (FC), determined gravimetrically. Plant height and trunk diameter were monitored throughout. Drought symptoms—including dry leaves and chlorosis—were assessed under both control (80% FC) and drought (40% FC) conditions. Severity was visually categorized as unaffected (green), partially affected (half symptomatic), or severely affected (fully dry). Controls helped distinguish stress symptoms from seasonal senescence (Figure S6). Each drought-treatment group included 40 replicates for water control, 32 for TSB/100, and 30 for inoculated treatments.

### 4.8. Statistical Analyses

All statistical analyses were conducted with a significance level of alpha = 0.05. Hazard ratios were reported with their corresponding 95% confidence interval and *p*-value.

### 4.9. Multivariate Analysis-Driven Selection of the Best Candidate Strains for SynComs Selection

Multiple correspondence analysis (MCA), an extension of correspondence analysis (Sobue et al., 2017 [99]), was used to assess relationships among several ordinal variables: PGPB traits, EPS production, tolerance to osmotic (PEG 10, PEG 30) and saline (NaCl 0.5–1.5 M) stress, soil depth (0–5 cm, 5–10 cm), isolation compartment (Root, RAS, non-RAS, RIM), and growth medium (TSA/10, TSA/20, TSA/50). Numerical variables were converted to ordinal scales based on performance ranges.

Principal Component Analysis (PCA) was also conducted to explore associations between SynComs/single strains and plant traits, including germination rate, post-germination development, root architecture, and biofilm formation. Data were normalized prior to PCA. Numerical variables were converted to ordinal scales based on performance ranges. In Figure 4, the lines representing iso-response values were generated using the script provided by Gabriel Zoppoli [100].

### 4.10. Statistical Analyses of Plant Symptoms and Growth Parameters

Bacterial strain growth parameters were analyzed using the R package Growthcurver (version 1.5.0) [101].

The proportion of germination and developed seedlings of the experiment with single strains and with consortia (synthetic communities) were analyzed with the Chi-Squared test, followed by the analysis of the standardized adjusted residuals in RStudio 2023.06.01 (running under R v4.4.2, R Core Team 2024, Vienna, Austria) using the native function chisq.test and the packages dplyr (v1.1.4) [102] and rstatix (v0.7.2) [103].

Principal component analysis (PCA) and Multiple Correspondence Analysis (MCA) were performed using the FactoMineR (v2.7) R package [104].

### 4.11. Survival Models

To evaluate the impact of bacterial inoculants on plant survival under drought, we combined several survival analysis methods. Kaplan–Meier survival curves illustrated the progression of survival probabilities across treatments, and the log-rank test assessed statistical differences between them.

To account for time-dependent variables, we used the Cox proportional hazards model, which estimated the relative mortality risk (hazard ratio, HR) for each treatment, adjusting for covariates like plant height and stem diameter. Hazard Ratio captures changes in risk over time. Multivariable survival analysis helped refine treatment-specific HR estimates by incorporating continuous predictors. The cumulative hazard function describes total mortality risk up to a given point, highlighting how risk accumulates over time.

### 4.12. Multinomial Logistic Regression

Multinomial logistic regression was used to assess the relationship between continuous variables (height and diameter increment, time of symptom observation) and the categorical variable Inoculant (SynCom and/or single strain) under drought conditions. The goal was to predict symptom development based on observed growth parameters after inoculation. Multinomial logistic regression was selected over ordinal regression to allow distinct sets of coefficients for each symptom category. This was essential, as drought was expected to influence the probabilities of each category differently (e.g., increasing ‘very affected’ while decreasing ‘not affected’), violating the proportional odds assumption of ordinal models. Model fit was evaluated using the Akaike Information Criterion (AIC), Nagelkerke’s R^2^ (R2N), and *p*-values. Relationships were expressed via odds ratios, log odds estimates and marginal means. Analyses were performed with the R package survminer (v0.5.0) [105] and Jamovi (v2.3) [106,107] for additional calculations and visualizations.

### 4.13. Use of GenAI

GenAI was used in the paper to draft the network in Figure S8 for literature mining and to improve text readability.

## 5. Conclusions

In this study, we developed a nature-based strategy for the production of drought-resilient tree seedlings to be used in restoration initiatives of the Mediterranean forests by designing soil-compartment–derived bacterial SynComs tailored to plant traits. By isolating strain along ecological (compartment and depth) and functional (PGPB traits) gradients, we showed that it is possible to assemble effective synthetic microbial communities that provide drought protection to host plants. Our strategy prioritized strains that thrive in distinct soil niches, thereby promoting niche complementarity, functional synergy, and likely persistence in the rhizosphere. The resulting consortia not only improved early seedling development in *Arabidopsis* but also conferred drought tolerance to two Mediterranean forest species, *Quercus pubescens* and *Sorbus domestica*.

One key practical message is that sampling design matters: combining spatially informed bacterial isolation with trait-based selection enables the development of targeted SynComs with high functional potential. Another one is that alignment between microbial traits and plant root strategies significantly improves SynCom efficacy. Our findings suggest that conservative root systems, such as those of *Q. pubescens*, benefit from EPS-producing consortia that reinforce soil structure and water retention, whereas acquisitive roots, such as those of *S. domestica*, respond better to hormone-producing consortia that boost rapid growth under stress.

These results offer a replicable framework for microbial-assisted reforestation and climate adaptation strategies. By integrating plant traits, soil microbial ecology, and multivariate screening approaches, our study provides both a methodological and conceptual basis for the ecological design of beneficial microbial consortia to support ecosystem restoration in drought-prone areas.

## Data Availability

Data are contained within the article and Supplementary Materials.

## Supplementary Materials

The following supporting information can be downloaded at: https://www.mdpi.com/article/10.3390/plants14111659/s1. Figure S1: K-means clustering of bacterial strain tolerance to osmotic stress; Figure S2: Growth Dynamics of Selected Bacterial Strains. Figure S3: Biofilm formation by individual strains and four-strain consortia. Figure S4: Germination and seedling development of *Arabidopsis thaliana* under inoculation with single strains (assay N°1; Figure S5: Germination and seedling development of *Arabidopsis thaliana* under inoculation with single strains (assay N°2); Figure S6: Germination and seedling development of *Arabidopsis thaliana* under inoculation with synthetic communities (SynCom); Figure S7: Development of drought-related symptoms in plants; Figure S8: Estimated probabilities of symptom development under the most efficient synthetic communities for each tree species; Figure S9: Similarity network of synthetic communities based on shared strain composition; Figure S10: Evaluation of biofilm synergy in synthetic consortia; Figure S11: Greenhouse experimental setup for tree seedling trials. Table S1: Bacterial strain consortia used in the biofilm assay; File S1: Strain traits; File S2: Syncom assembly.
