# Supplementary material for: Soil-Gradient-Derived Bacterial Synthetic Communities Enhance Drought Tolerance in Quercus pubescens and Sorbus domestica Seedlings
Source: Plants (Basel). 2025 May 29;14(11):1659. doi: 10.3390/plants14111659 (PMC12158234; doi:10.3390/plants14111659)
Supplement: Supplementary file 1 [file plants-14-01659-s001.zip › Figures_supp_v02.pdf]

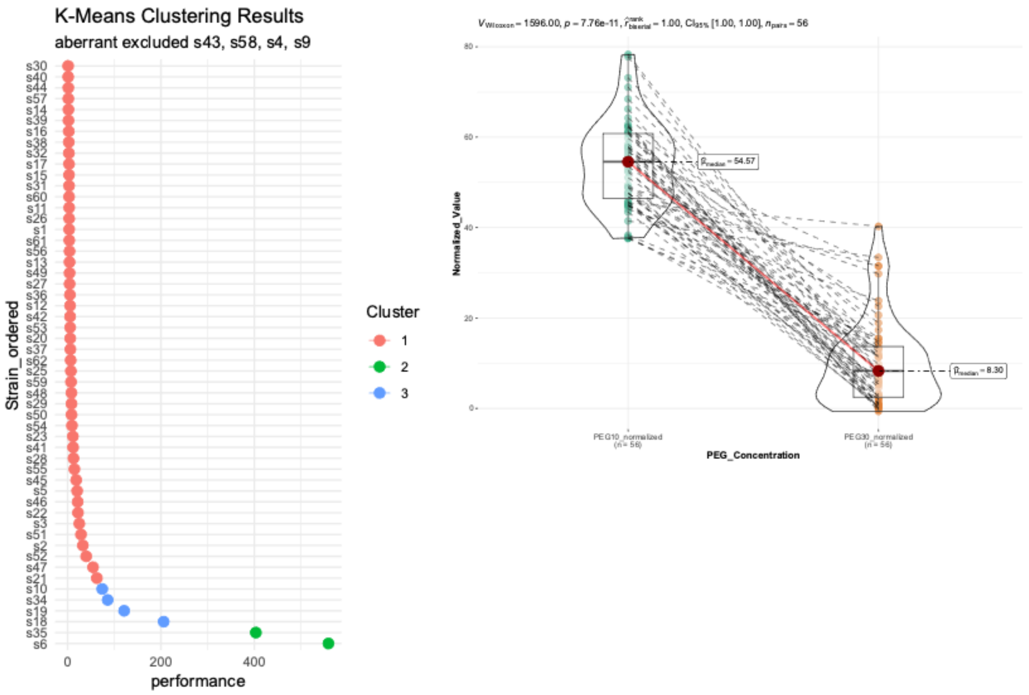

Figure S1. K-means clustering of bacterial strain tolerance to osmotic stress.

Left: Strains were grown in 10-fold diluted Tryptic Soy Broth supplemented with polyethylene glycol (PEG6000) at 10%, 20%, and 30%, corresponding to osmotic potentials of −0.51, −1.23, and −2.56 MPa, respectively. Bacterial growth was monitored by OD_{590nm} measurements (Infinite® M1000 plate reader, Tecan) and converted to CFU mL^{-1} using established calibration curves. Strain classification into sensitive, tolerant, or resistant groups was performed using k-means clustering based on a performance index (P), calculated as the normalized CFU/mL at PEG10 and PEG30 relative to control conditions: Performance (P) = PEG10 / PEG30. Most strains (91.07%) were tolerant or resistant to osmotic stress. Strains s43, s58, s4, and s9, which failed to grow at -1.23 MPa, were excluded from the panel.

Right: Effect of osmotic stress on bacterial growth expressed as normalized CFU mL^{-1} at different PEG concentrations (PEG6000) at 10%, and 30%, corresponding to osmotic potentials of −0.51, and −2.56. Growth significantly declined at higher osmotic potential, with a Wilcoxon test confirming a strong difference between 10% and 30% PEG (p-value = 7.5×10^{-11}).

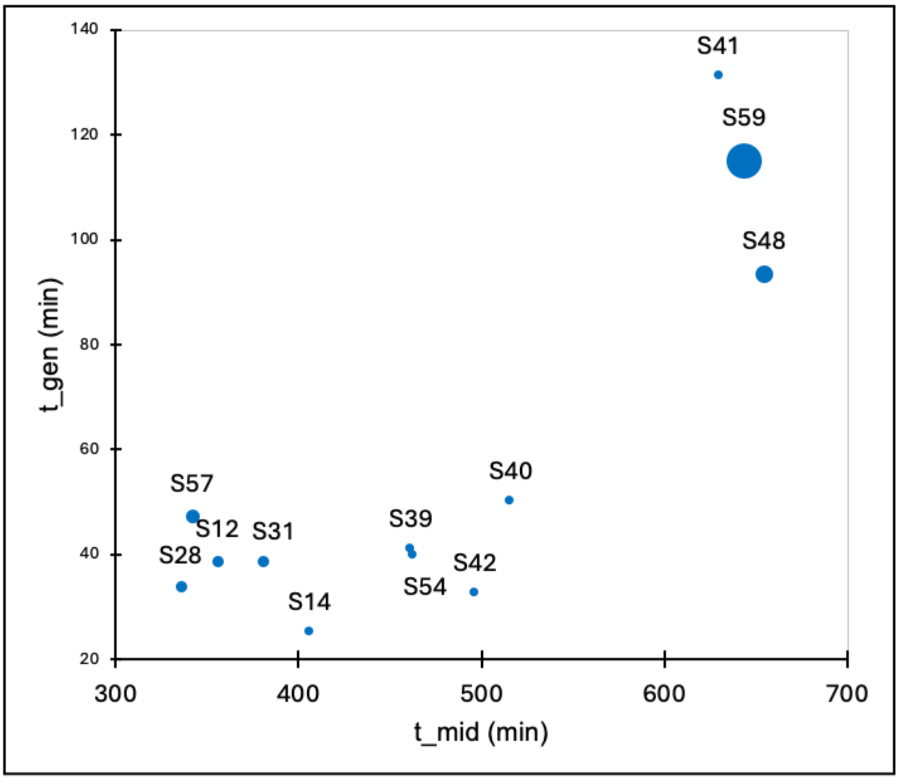

Figure S2. Growth Dynamics of Selected Bacterial Strains.
Generation time (t_gen) *vs.* half-carrying capacity (t_mid) for the twelve selected strains in tryptic soy broth (TSB) ten-fold diluted, at 30°C. t_mid is the time at which the population density reaches half of the Carrying Capacity $(K/2)$ that occurs at the inflection point of the growing curve; t_gen is the fastest possible generation time, called the doubling time. Population size N0 at the beginning of the growth curve is illustrated by the diameter of the dot.

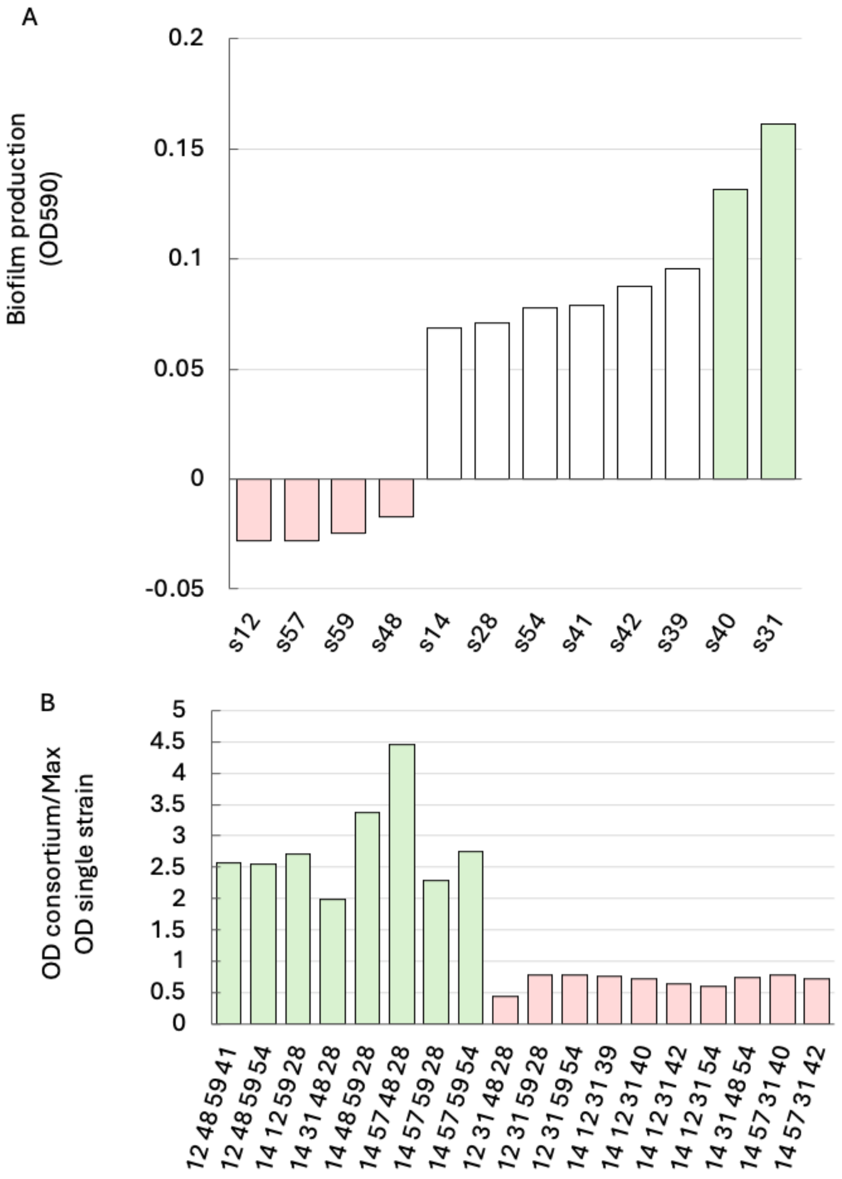

Figure S3. Biofilm formation by individual strains and four-strain consortia. A) Biofilm production (OD_{590}) of the twelve individual strains used in the assay. Bars are color-coded to indicate biofilm capacity: pink (non-producers), white (intermediate producers), and green (strong producers). B) Biofilm synergy index: ratio of each consortium's OD_{590} value to the highest OD_{590} value of its best-performing single strain. Green bars indicate consortia with enhanced biofilm production (ratio > 1.9), while pink bars show reduced performance (ratio < 0.8). Error bars are not shown, as the assay was repeated three times under slightly varying conditions (e.g., initial cell density, incubation time, duration of the biofilm staining with crystal violet), but with only a single replicate in each run. The aim was to explore optimal conditions for biofilm formation across all strains. This assay is well-established and known to be highly reproducible (Ren et al., 2013). To ensure comparability, we chose to screen all strains and consortia on the same 96-well plate. Despite the slight variations between experiments, the relative hierarchy of biofilm production remained consistent.

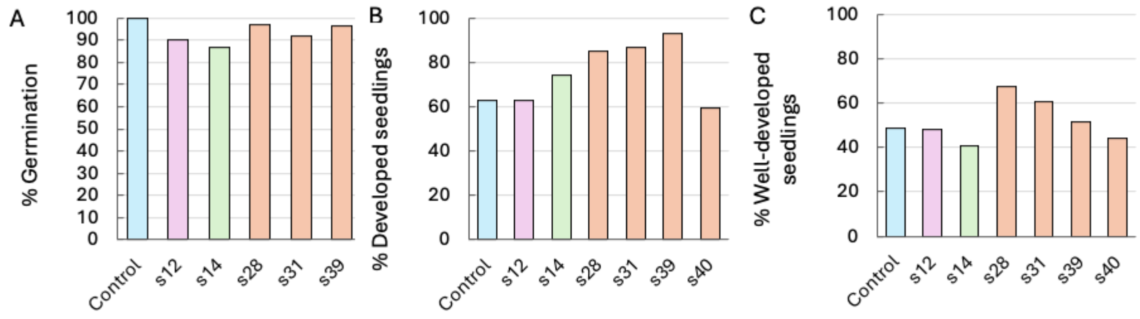

Figure S4. Germination and seedling development of *Arabidopsis thaliana* under inoculation with single strains (assay N°1). A) Percentage of germination of seeds. B) percentage (out of the germinated) of developed seedlings. C) percentage of well-developed seedlings of *Arabidopsis thaliana* non-inoculated (control, pale blue bar) or inoculated with different single strains: s12 – *Peribacillus simplex*, s14 – *Pantoea pleuroti*, s28 – *Pseudomonas umsongensis*, 31 – *Pseudomonas migulae*, 39 – *Pseudomonas silesiensis*. Pink bars: strains with high EPS production and strong osmotic tolerance; green bars: strains with high auxin (IAA) production and strong osmotic tolerance; orange bars: strains with intermediate levels of IAA production and osmotic tolerance. n = 35 seeds (control, strain 28), n = 30 seeds (strains 12, 14, 39, and 40), n = 25 seeds (strain 31). The asterisk denotes significant differences (p-value< 0.05) according to a Chi Square test of Independence.

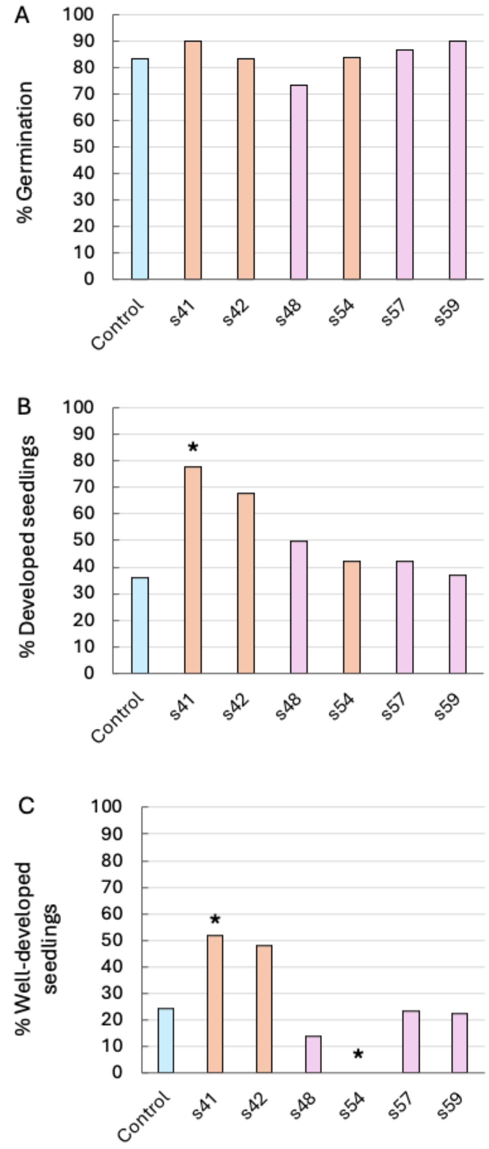

Figure S5. Germination and seedling development of *Arabidopsis thaliana* under inoculation with single strains (assay N°2).
A) Percentage of germination of seeds. B), percentage (out of the germinated) of developed seedlings. C) Percentage of well-developed seedlings of Arabidopsis non-inoculated (control, pale blue bar) or inoculated with different single strains: s41: *Pseudomonas* sp., s42 - *Pseudomonas silesiensis*, s48 - *Caballeronia glathei*, s54 - *Pseudomonas mandelii*, s57 - *Peribacillus simplex*, s59 - *Pseudomonas lini*, s28 - *Pseudomonas umsongensis*. Pink bars: strains with high EPS production and osmotic tolerance, orange bars: strains with intermediary IAA production and osmotic tolerance. The asterisk denotes significant difference according to the Chi Square test of Independence. n = 30 seeds (control, strains 41, 42, 48, 57, and 59), n = 31 seeds (strain 54).

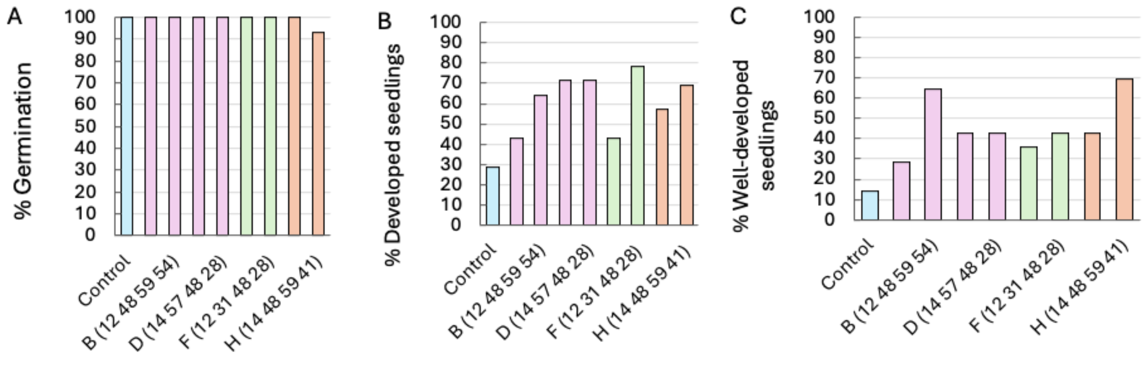

Figure S6. Germination and seedling development of *Arabidopsis thaliana* under inoculation with synthetic communities (SynCom). A) Percentage of seed germination. B) Percentage of germinated seeds that developed into seedlings. C) Percentage of well-developed seedlings among the germinated ones, comparing non-inoculated controls (blue bar) with plants inoculated with various synthetic microbial consortia (SynComs).

SynComs are color-coded based on their biofilm production capacity: pink = high, orange = intermediate, green = low. Strains used in the SynComs: s12 – *Peribacillus simplex*, s14 – *Pantoea pleuroti*, s28 – *Pseudomonas umsongensis*, 31 – *Pseudomonas migulae*, 39 – *Pseudomonas silesiensis*, s41 – *Pseudomonas* sp., s42 – *Pseudomonas silesiensis*, s48 – *Caballeronia glathei*, s54 – *Pseudomonas mandelii*, s57 – *Peribacillus simplex*, s59 – *Pseudomonas lini*.

n = 14 seeds per treatment. The asterisk denotes significant differences (p-value< 0.05) according to a Chi Square test of Independence.

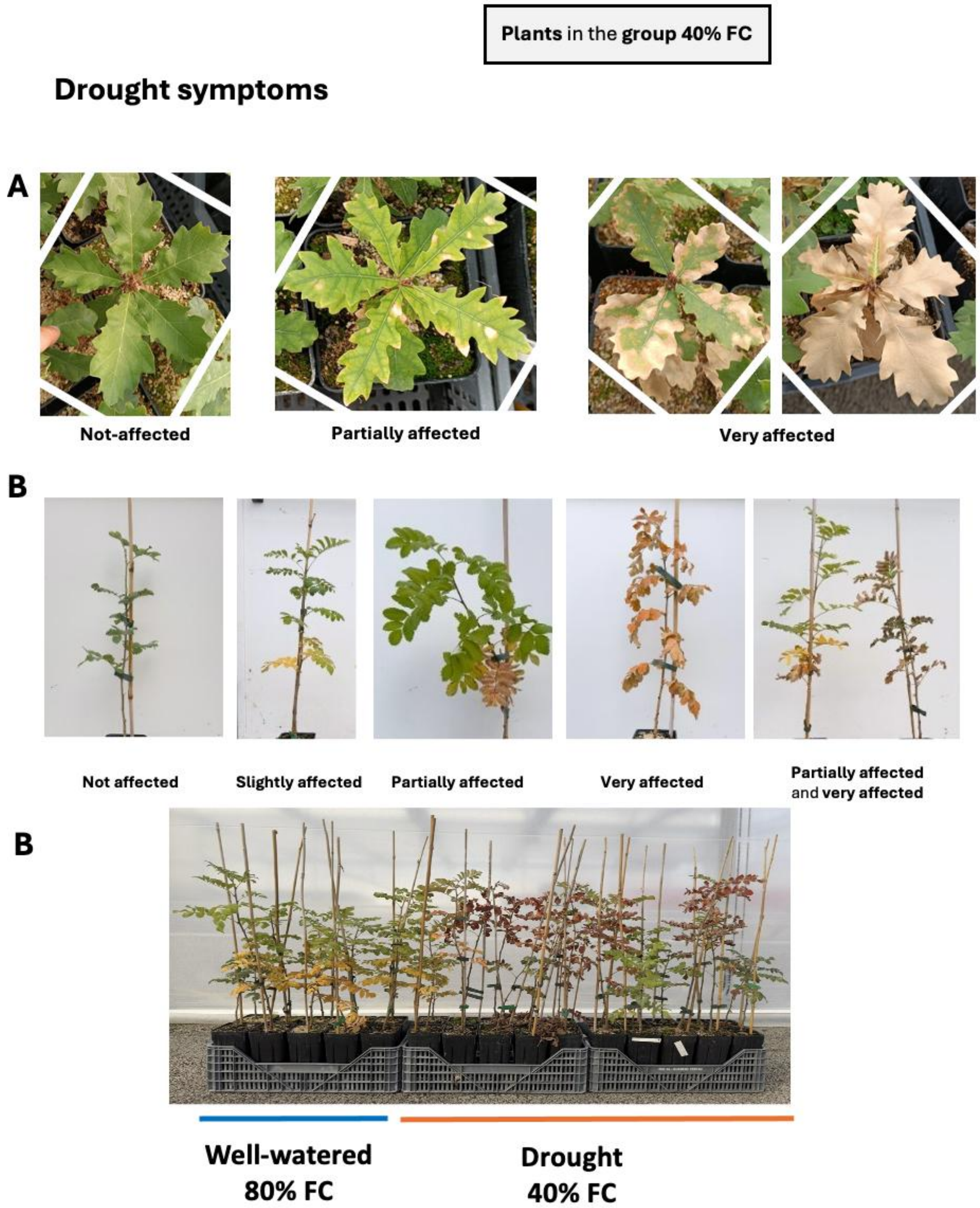

Figure S7. Development of drought-related symptoms in plants. A) *Quercus pubescens*. B) *Sorbus domestica*. C) At the end of the experiment, well-watered control plants (left) displayed natural signs of senescence typical of autumn onset, while drought-treated plants (right) exhibited clear symptoms of water stress.

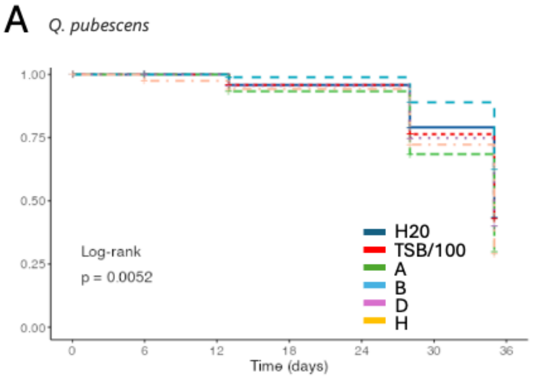

Cox Table- Treatment *Q. pubescens*

Explanatory	Levels	all	HR (Univariable)
TreatmentBac	11-H2O	200 (20.8)	-
	16-TSB/100	160 (16.7)	1.05 (0.65-1.69, p=0.843)
	A (12 48 59 41)	150 (15.6)	1.50 (0.96-2.35, p=0.078)
	B (12 48 59 54)	150 (15.6)	0.54 (0.30-0.97, p=0.039)
	D (14 57 48 28)	150 (15.6)	1.13 (0.70-1.83, p=0.605)
	H (14 48 59 41)	150 (15.6)	1.46 (0.93-2.30, p=0.099)

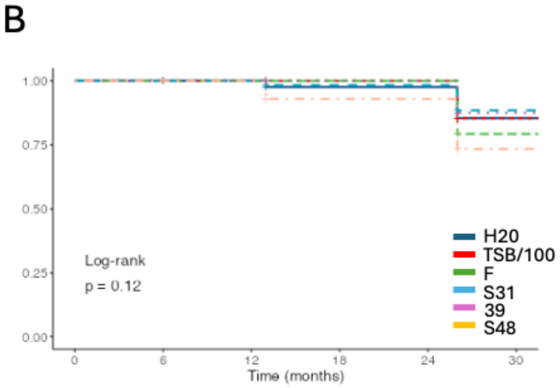

Cox Table- Treatment *Q. pubescens*

Explanatory	Levels	all	HR (Univariable)
TreatmentBac	21-H2O	112 (20.0)	-
	26-TSB/100	96 (17.1)	0.97 (0.48-1.96, p=0.925)
	F (12 31 48 28)	116 (20.7)	1.45 (0.78-2.69, p=0.245)
	S31	80 (14.3)	0.72 (0.32-1.61, p=0.422)
	S39	80 (14.3)	1.11 (0.54-2.28, p=0.780)
	S48	76 (13.6)	1.85 (0.97-3.53, p=0.062)

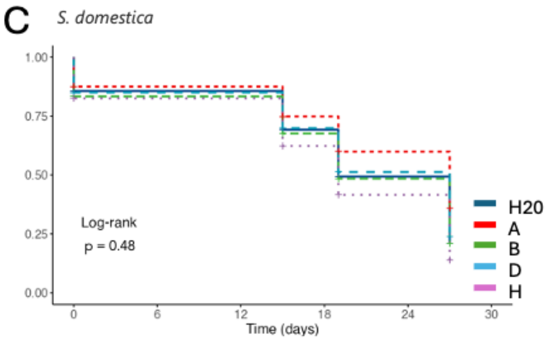

Cox Table- Treatment *S. domestica*

Explanatory	Levels	all	HR (Univariable)
Treatment	11-H20	160 (25.0)	-
	A (12 48 59 41)	120 (18.8)	0.71 (0.51-1.00, p=0.051)
	B (12 48 59 54)	120 (18.8)	1.03 (0.76-1.40, p=0.853)
	D (14 57 48 28)	120 (18.8)	0.94 (0.69-1.30, p=0.725)
	H (14 48 59 41)	120 (18.8)	1.25 (0.93-1.68, p=0.141)

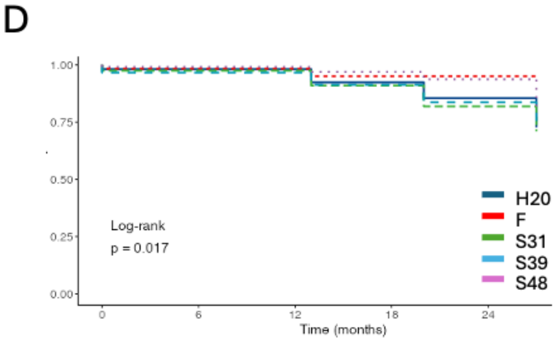

Cox Table- Treatment *S. domestica*

Explanatory	Levels	all	HR (Univariable)
Treatment	21-H2O	160 (25.0)	-
	F (12 31 48 28)	120 (18.8)	0.29 (0.11-0.77, p=0.013)
	S31	120 (18.8)	1.15 (0.63-2.13, p=0.646)
	S39	120 (18.8)	1.09 (0.58-2.03, p=0.785)
	S48	120 (18.8)	0.54 (0.25-1.17, p=0.117)

Figure S8: Estimated probabilities of symptom development under the most efficient synthetic communities for each tree species. A) *Quercus pubescens*. B) *Sorbus domestica*. Probabilities were calculated using estimated marginal means from a multinomial logistic regression model based on observed symptom categories.

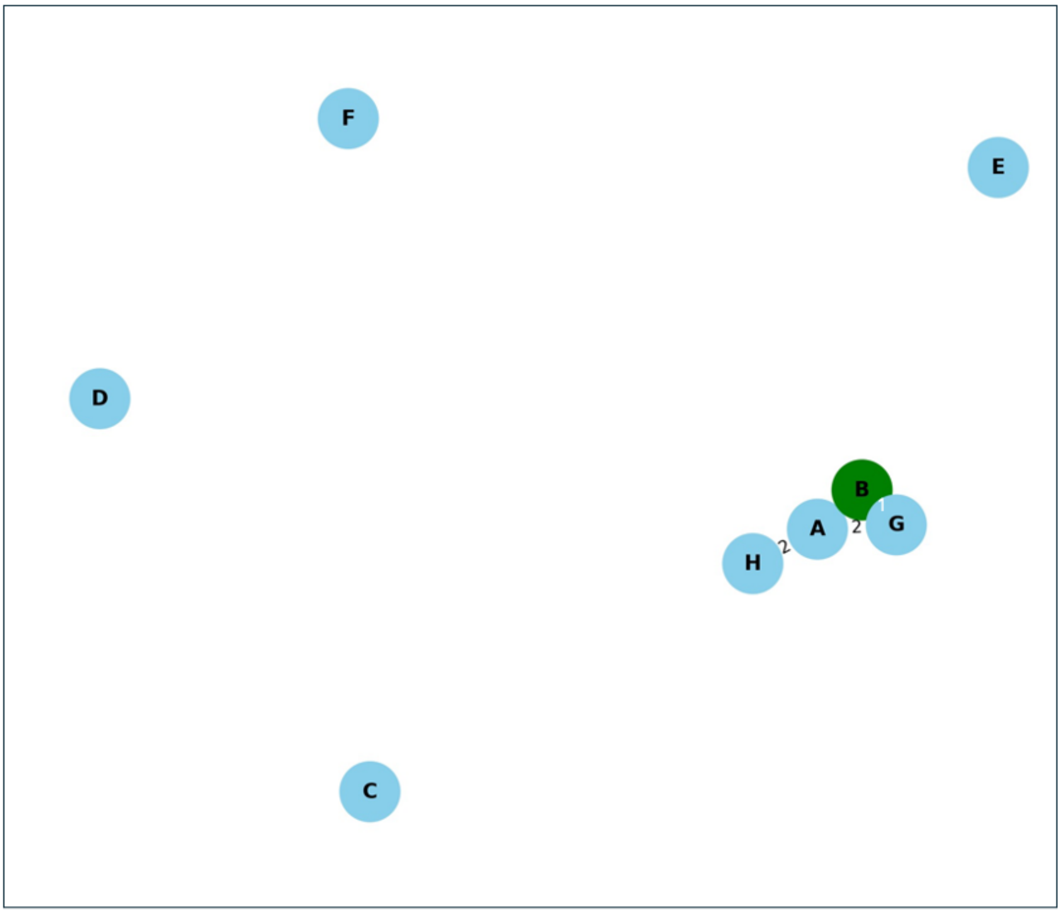

Figure S9: Similarity network of synthetic communities based on shared strain composition. Edges connect SynComs that differ by a single strain, illustrating compositional proximity. Figure generated with GenAI.

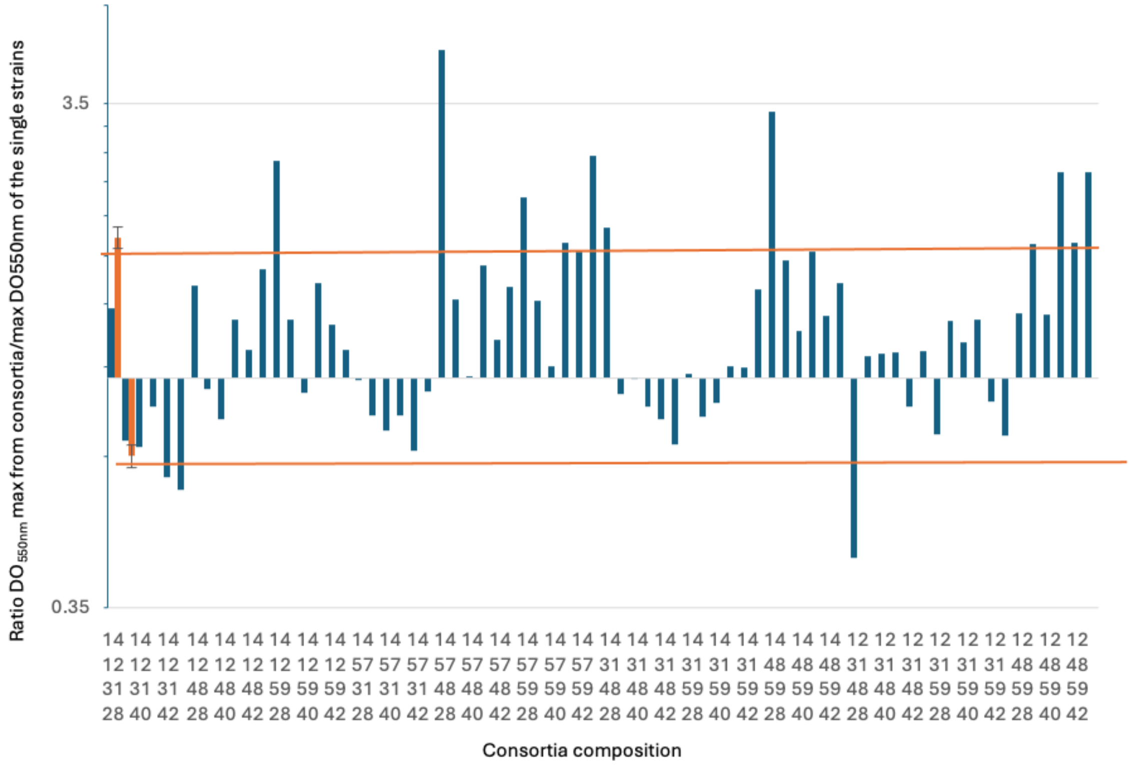

Figure S10. Evaluation of biofilm synergy in synthetic consortia. Synergy was assessed by calculating the ratio of each consortium OD_{590} to the highest OD_{590} observed among its individual strains. A ratio $\geq$ 1.9 indicated synergistic biofilm formation, while a ratio < 0.7 suggested antagonistic effects. Intermediate values reflected minor differences in performance. These thresholds (0.7 and 1.9) were empirically determined (see Figure S9) to identify a subset of consortia for further testing. Red lines represent the critical synergy/antagonism thresholds.

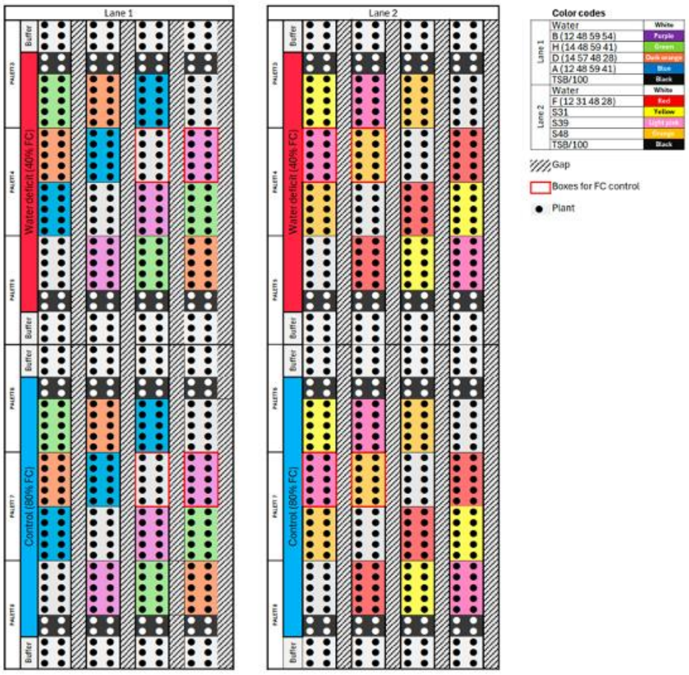

Figure S11. Greenhouse experimental setup for tree seedling trials. The setup, identical for *Quercus pubescens* and *Sorbus domestica* seedlings, followed a randomized complete block design to minimize environmental heterogeneity within the greenhouse. Each block included all treatments and controls, allowing for within-block comparisons and reducing spatial variation effects. Plants were irrigated using an automated system simulating natural rainfall. Drought-treated plants (40% field capacity) and well-watered plants (80% field capacity) were separated by two rows of buffer plants, which also bordered the north and south edges of the setup.

Table S1. Bacterial strain consortia used in the biofilm assay. Each number corresponds to an individual bacterial strain. S12 – *Peribacillus simplex*, s14 – *Pantoea pleuroti*, s28 – *Pseudomonas umsongensis*, s31 – *Pseudomonas migulae*, s39 – *Pseudomonas silesiensis*, s41 – *Pseudomonas* sp.,s 42 – *Pseudomonas silesiensis*, s48 – *Caballeronia glathei*, s54 – *Pseudomonas mandelii*, s57 – *Peribacillus simplex*, s59 – *Pseudomonas lini*.

14 12 31 28	14 12 31 39	14 12 31 40	14 12 31 41	14 12 31 42	14 12 31 54
14 12 48 28	14 12 48 39	14 12 48 40	14 12 48 41	14 12 48 42	14 12 48 54
14 12 59 28	14 12 59 39	14 12 59 40	14 12 59 41	14 12 59 42	14 12 59 54
14 57 31 28	14 57 31 39	14 57 31 40	14 57 31 41	14 57 31 42	14 57 31 54
14 57 48 28	14 57 48 39	14 57 48 40	14 57 48 41	14 57 48 42	14 57 48 54
14 57 59 28	14 57 59 39	14 57 59 40	14 57 59 41	14 57 59 42	14 57 59 54
14 31 48 28	14 31 48 39	14 31 48 40	14 31 48 41	14 31 48 42	14 31 48 54
14 31 59 28	14 31 59 39	14 31 59 40	14 31 59 41	14 31 59 42	14 31 59 54
14 48 59 28	14 48 59 39	14 48 59 40	14 48 59 41	14 48 59 42	14 48 59 54
12 31 48 28	12 31 48 39	12 31 48 40	12 31 48 41	12 31 48 42	12 31 48 54
12 31 59 28	12 31 59 39	12 31 59 40	12 31 59 41	12 31 59 42	12 31 59 54
12 48 59 28	12 48 59 39	12 48 59 40	12 48 59 41	12 48 59 42	12 48 59 54
